# Structure, Spin Correlations, and Magnetism of the *S* = 1/2 Square-Lattice Antiferromagnet Sr_2_CuTe_1–*x*_W_*x*_O_6_ (0 ≤ *x* ≤ 1)

**DOI:** 10.1021/acs.chemmater.3c02535

**Published:** 2023-12-25

**Authors:** Otto H. J. Mustonen, Ellen Fogh, Joseph A. M. Paddison, Lucile Mangin-Thro, Thomas Hansen, Helen Y. Playford, Maria Diaz-Lopez, Peter Babkevich, Sami Vasala, Maarit Karppinen, Edmund J. Cussen, Henrik M. Ro̷nnow, Helen C. Walker

**Affiliations:** † School of Chemistry, University of Birmingham, Birmingham B15 2TT, United Kingdom; ‡ Department of Material Science and Engineering, University of Sheffield, Sheffield S1 3JD, United Kingdom; § Laboratory for Quantum Magnetism, Institute of Physics, École Polytechnique Fédérale de Lausanne (EPFL), Lausanne CH-1015, Switzerland; ∥ Materials Science and Technology Division, Oak Ridge National Laboratory, Oak Ridge, Tennessee 37831, United States; ⊥ Institut Laue Langevin, 71 Avenue des Martyrs, CS 20156, Grenoble Cedex 9 F-38042, France; # ISIS Neutron and Muon Source, Rutherford Appleton Laboratory, Chilton, Didcot OX11 OQX, United Kingdom; ∇ CNRS, Grenoble INP, Institut Néel, Université Grenoble Alpes, Grenoble 38000, France; ○ ESRF - The European Synchrotron, Grenoble 38000, France; ◆ Department of Chemistry and Materials Science, Aalto University, Espoo FI-00076, Finland

## Abstract

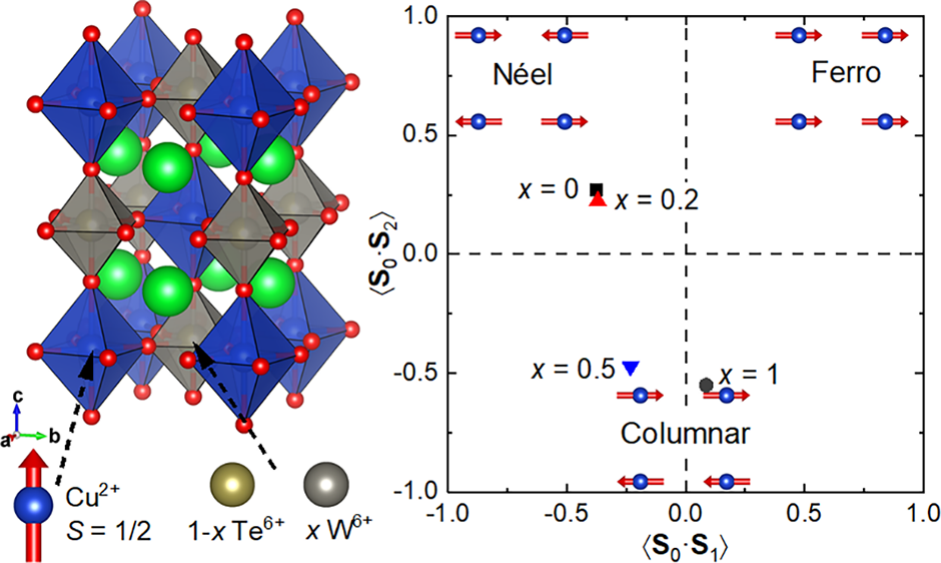

Quantum spin liquids are highly entangled magnetic states with exotic properties. The *S* = 1/2 square-lattice Heisenberg model is one of the foundational models in frustrated magnetism with a predicted, but never observed, quantum spin liquid state. Isostructural double perovskites Sr_2_CuTeO_6_ and Sr_2_CuWO_6_ are physical realizations of this model but have distinctly different types of magnetic order and interactions due to a d^10^/d^0^ effect. Long-range magnetic order is suppressed in the solid solution Sr_2_CuTe_1–*x*_W_*x*_O_6_ in a wide region of *x* = 0.05–0.6, where the ground state has been proposed to be a disorder-induced spin liquid. Here, we present a comprehensive neutron scattering study of this system. We show using polarized neutron scattering that the spin liquid-like *x* = 0.2 and *x* = 0.5 samples have distinctly different local spin correlations, which suggests that they have different ground states. Low-temperature neutron diffraction measurements of the magnetically ordered W-rich samples reveal magnetic phase separation, which suggests that the previously ignored interlayer coupling between the square planes plays a role in the suppression of magnetic order at *x* ≈ 0.6. These results highlight the complex magnetism of Sr_2_CuTe_1–*x*_W_*x*_O_6_ and hint at a new quantum critical point between 0.2 < *x* < 0.4.

## 1 Introduction

Spin-1/2 square-lattice antiferromagnets have been of significant scientific interest since the discovery of high-*T*_c_ superconductivity in cuprates.^1^ These antiferromagnetic and insulating parent phases become superconducting upon hole or electron doping.^2^ The *S* = 1/2 square-lattice Heisenberg model is also one of the foundational models of frustrated magnetism.^3^ This model has two magnetic interactions: the nearest-neighbor *J*_1_ interaction along the side of the square and the next-nearest-neighbor *J*_2_ along the diagonal. A dominant antiferromagnetic *J*_1_ leads to Néel antiferromagnetic order as observed in the high-*T*_c_ parent phases, while dominant *J*_2_ leads to columnar antiferromagnetic order. The competition between antiferromagnetic *J*_1_ and *J*_2_ interactions leads to magnetic frustration, which is predicted to stabilize a quantum spin liquid in the highly frustrated *J*_2_/*J*_1_ ≈ 0.4–0.6 region.^3−10^ Quantum spin liquids are exotic quantum states consisting of highly entangled spins, which remain dynamic even at absolute zero without magnetic order or spin freezing.^11−13^ A number of *S* = 1/2 square-lattice antiferromagnets are known with either Néel or columnar antiferromagnetic order,^14−20^ but the predicted quantum spin liquid state has never been observed. However, recent theoretical studies propose disorder as a possible route for stabilizing a spin liquid-like state.^21−25^

**Figure 1:**
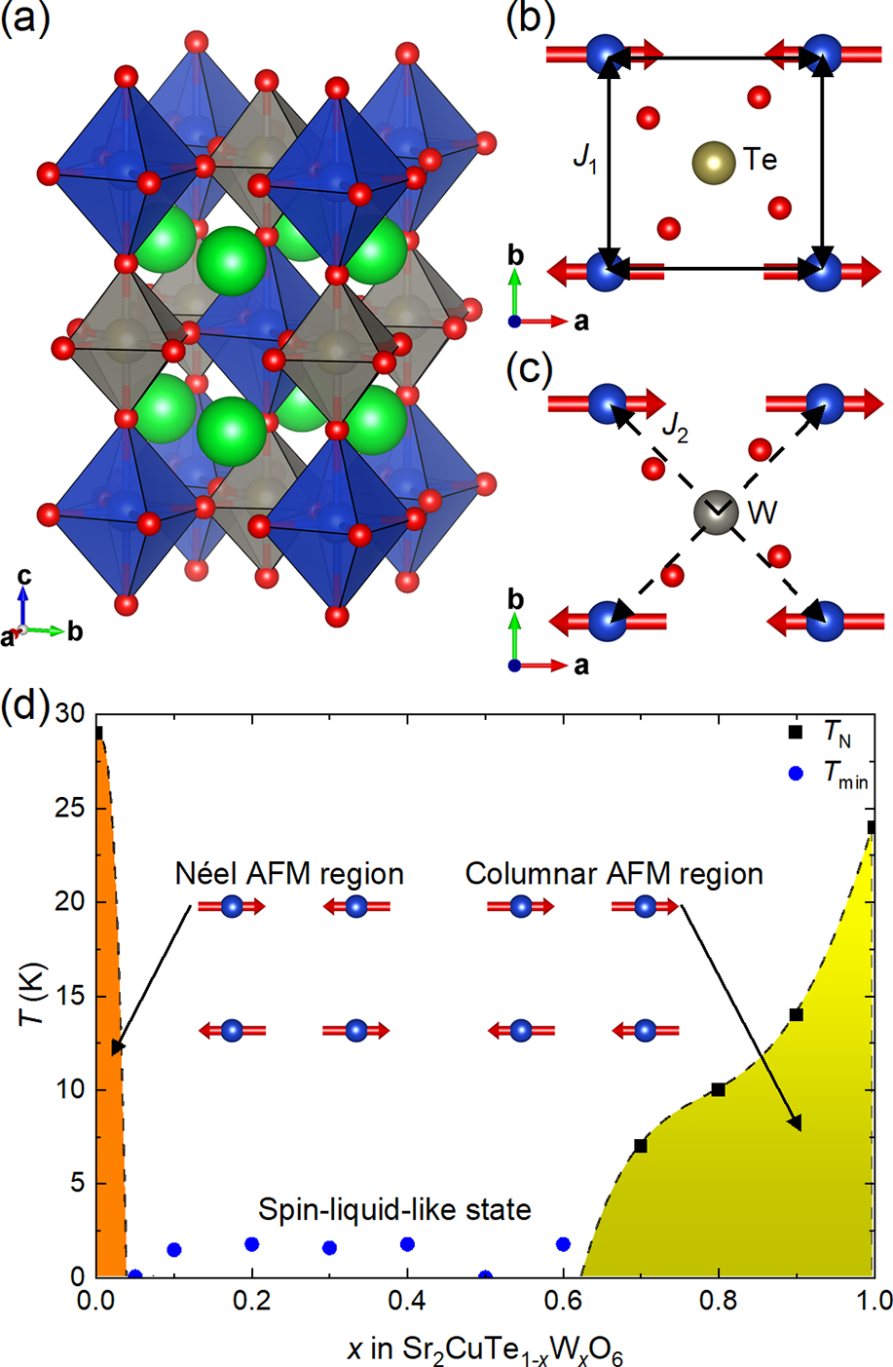
(a) *B*-site ordered double perovskite structure of Sr_2_CuTe_1–*x*_W_*x*_O_6_, where the green, blue, gray, and red spheres represent Sr, Cu, Te/W, and O, respectively. The magnetic interactions are highly two-dimensional in the *ab* plane, where the *S* = 1/2 Cu^2+^ cations form a square lattice. (b) Néel antiferromagnetic structure of Sr_2_CuTeO_6_ (*x* = 0) stabilized by a dominant antiferromagnetic *J*_1_ interaction. (c) Columnar magnetic structure of Sr_2_CuWO_6_ (*x* = 1) stabilized by a dominant antiferromagnetic *J*_2_ interaction. (d) Magnetic phase diagram of the solid solution Sr_2_CuTe_1–*x*_W_*x*_O_6_ based on muon spectroscopy from refs (48) and (50). The black squares represent measured Néel temperatures, and the blue circles represent the lowest temperatures measured, where the magnetism remains dynamic. A spin liquid-like state without magnetic order is observed between *x* = 0.05 and *x* = 0.6.

The *B*-site ordered double perovskites Sr_2_CuTeO_6_ and Sr_2_CuWO_6_ are excellent realizations of the *S* = 1/2 square-lattice Heisenberg model.^26,27^ These compounds crystallize in the tetragonal space group *I*4/*m* with complete rocksalt ordering of the Cu^2+^ and Te^6+^/W^6+^ cations on the octahedral *B′*- and *B″-*sites as shown in Figure 1a.^28−30^ The magnetic interactions in these materials are highly two-dimensional in the *ab*-plane due to a Jahn–Teller distortion and co-operative orbital ordering of the 3d^9^*S* = 1/2 Cu^2+^ cations.^30−33^ Remarkably, despite being isostructural, Sr_2_CuTeO_6_ and Sr_2_CuWO_6_ have distinctly different magnetic interactions and ground states. Sr_2_CuTeO_6_ has the Néel antiferromagnetic structure (Figure 1b) below *T*_N_ = 29 K with a propagation vector of **k** = (1/2, 1/2, 0).^34^ This structure is stabilized by the strong nearest-neighbor *J*_1_ interaction with *J*_1_ = −7.18 meV and *J*_2_ = −0.21 meV.^26^ In contrast, Sr_2_CuWO_6_ has the columnar antiferromagnetic structure below *T*_N_ = 24 K with **k** = (0, 1/2, 1/2) (see Figure 1c).^35^ This structure is stabilized by the strong next-nearest-neighbor *J*_2_ interaction with *J*_1_ = −1.2 meV and *J*_2_ = −9.5 meV.^27^ The contrasting magnetic interactions of Sr_2_CuTeO_6_ and Sr_2_CuWO_6_ arise from differences in orbital hybridization of the 4d^10^ Te^6+^ and 5d^0^ W^6+^ cations. Strong W 5d–O 2p hybridization in Sr_2_CuWO_6_ enables the dominant Cu–O–W–O–Cu superexchange along the diagonal *J*_2_.^27,36^ Conversely, the dominant *J*_1_ interaction in Sr_2_CuTeO_6_ is a Cu–O–O–Cu superexchange without significant participation from the Te 4d^10^ states.^26^ This d^10^/d^0^ effect is general for magnetic 3d transition metal double perovskites^37,38^ including other Cu^2+^ systems.^39−46^

Given that Sr_2_CuTeO_6_ (strong *J*_1_) and Sr_2_CuWO_6_ (strong *J*_2_) are located on the opposite sides of the predicted *J*_2_/*J*_1_ ≈ 0.4–0.6 spin liquid region in the *S* = 1/2 square-lattice Heisenberg model, the solid solution Sr_2_CuTe_1–*x*_W_*x*_O_6_ appeared like a natural system to look for the missing quantum spin liquid.^36^ Promisingly, a spin liquid-like state was first observed in the composition *x* = 0.5, where muon spectroscopy measurements revealed dynamic magnetism down to 19 mK.^47^ No evidence of spin freezing was found in the muon experiment nor in AC susceptibility measurements. A *T*-linear term was observed in the low-temperature specific heat, which is typical of spin liquids. Building on this work, the full Sr_2_CuTe_1–*x*_W_*x*_O_6_ magnetic phase diagram has been explored (Figure 1d).^48−51^ The Néel ordered state is only observed for *x* = 0–0.02.^34,51^ The spin liquid-like state is observed in the wide region of *x* = 0.05–0.6,^47−51^ which is incompatible with the very narrow region of stability expected for the *S* = 1/2 square-lattice quantum spin liquid. Finally, columnar magnetic order is found for *x* = 0.7–1.

The suppression of magnetic order in such a wide region in Sr_2_CuTe_1–*x*_W_*x*_O_6_ is likely related to the significant Te/W disorder on the *B″*-site. This results in a special type of bond disorder, where the *J*_1_ and *J*_2_ interactions are effectively switched on and off depending on the local *B″*-cation in the middle of each Cu^2+^ square.^52,53^ The ground state in the spin liquid-like region has been proposed to be a random-singlet state.^23−25,54,55^ The random-singlet state is a disorder-induced spin liquid, where spin singlets of different strengths are formed based on the underlying quenched disorder.^23^ Partial spin freezing into either a “spin jam” state^56^ or patches of Néel and columnar-type correlated spins^53^ have also been proposed for this region.

Here, we present an average and local scattering investigation of Sr_2_CuTe_1–*x*_W_*x*_O_6_, revealing new insights into this unique magnetic system. Using neutron diffraction, we show that the average crystal structure of *x* = 0.5 with the spin liquid-like state is tetragonal at low temperatures, retaining an undistorted square of *S* = 1/2 Cu^2+^ cations. Our combined neutron and synchrotron X-ray total scattering experiments show that the local structure of *x* = 0.5 is well described by the average structure. However, we are unable to resolve effects of any potential clustering or ordering of Te and W on the *B″*-site. Our neutron diffraction study of the magnetic order in *x* = 0.9, 0.8, and 0.7 reveals columnar magnetic order with **k** = (0, 1/2, 1/2) as expected. However, an additional reflection belonging to the propagation vector **k** = (0, 1/2, 0) is observed for *x* = 0.8 and 0.7. This suggests that Te-doping in the columnar region disturbs the interlayer magnetic interactions and is responsible for the suppression of magnetic order at *x* ≈ 0.6. Finally, our polarized neutron study of the compositions *x* = 0.2 and 0.5 reveals distinctly different local spin correlations related to the short-range correlated states above *T*_N_ in the parent phases. This suggests that *x* = 0.2 and 0.5 have different ground states despite both being in the spin-liquid-like region, and a quantum critical point is expected between 0.2 < *x* < 0.4.

## 2 Experimental Methods

Polycrystalline powder samples of Sr_2_CuTe_1–*x*_W_*x*_O_6_ with 0 ≤ *x* ≤ 1 were synthesized using a conventional solid-state synthesis method as described previously.^48^ Stoichiometric quantities of SrCO_3_, CuO, TeO_2_, and WO_3_ (≥99.995%, Alfa Aesar) were ground in an agate mortar. The precursor mixture was calcined in air at 900 °C for 12 h. Synthesis was carried out in air at 1050 °C for 72 h with intermittent grindings.

Time-of-flight neutron total scattering experiments were carried out at the POLARIS diffractometer^57^ at the ISIS Neutron and Muon Source. Approximately 11 g of Sr_2_CuTe_0.5_W_0.5_O_6_ (*x* = 0.5) powder was sealed in an 8 mm vanadium can. Experiments were carried out in a cryostat at temperatures of 5, 100, and 300 K. The empty sample can and cryostat were also measured for background correction. The data were reduced using standard procedures in Mantid^58^ to obtain the Bragg scattering patterns for individual detector banks. Rietveld refinement^59^ was carried out using FULLPROF,^60^ and the structural figures were made with VESTA.^61^ Synchrotron X-ray total scattering experiments were carried out at the I15-1 diffractometer at Diamond Light Source using a wavelength of 0.161669 Å (76 keV). A 2D PerkinElmer XRD4343CT detector was positioned 20 cm away from the capillary to maximize the Q-range for optimal pair-distribution function data quality. A dark (without X-rays) detector image was collected to determine the dark current contribution and subsequently subtracted from the data; the detector was kept in a constant read-out state and air-cooled with fans to maintain a constant temperature, which led to negligible changes to the dark current contribution during the experiment.

The pair-distribution functions (PDFs) were obtained using the GUDRUN software package. Neutron data collected at 300 K on POLARIS were corrected for absorption, multiple scattering, and background from the sample environment. X-ray scattering data collected at room temperature on I15-1 were corrected for scattering from the empty capillary, Compton scattering, and incident beam polarization. The neutron PDF was obtained with a maximum wavevector magnitude *Q*_max_ = 40 Å^–1^, and the X-ray PDF was obtained with *Q*_max_ = 25 Å^–1^. The PDFs were expressed as *D*(*r*), in the notation of ref (62).

Constant-wavelength neutron diffraction was measured at the high-intensity D20 diffractometer at the Institut Laue-Langevin. Two grams of Sr_2_CuTe_1–*x*_W_*x*_O_6_ powders with *x* = 0.7, 0.8, and 0.9 was enclosed in 6 mm vanadium cans. These compositions are known to magnetically order at *T*_N_ = 7, 11, and 15 K, respectively.^48^ The data were collected at a wavelength of 2.41 Å at temperatures of 2 and 30 K. The exact wavelength was determined by refining room-temperature neutron diffraction data against laboratory X-ray data for the *x* = 0.9 composition. Magnetic Bragg scattering was extracted by subtracting the nuclear scattering observed at 30 K from the 2 K data. The magnetic structure of the *x* = 0.9 sample was refined using FULLPROF.^60^ Potential **k**-vectors in the *I*4/*m* space group were considered based on the Brillouin zone database^63^ of the Bilbao Crystallographic Server^64−66^ and the k-search program included in the FULLPROF Suite.^60^ Magnetic phase fractions for *x* = 0.8 and 0.7 were evaluated by refining the two main magnetic peaks while fixing the ordered moment to be the same in both magnetic phases. The scale factor was fixed by first refining the crystal structure of the corresponding sample. The full width at half-maximum (fwhm) was evaluated by fitting a single peak Voigt function.

Diffuse magnetic scattering was investigated at the D7 diffuse scattering spectrometer^67,68^ at the Institut Laue-Langevin. Sample powder (11–19 g) was sealed in aluminum cans with inserts to form an annulus shape. The samples were measured using cold neutrons with a wavelength of 4.8 Å (*E*_i_ = 3.55 meV). An Orange cryostat was used for temperature control. The samples Sr_2_CuTeO_6_ (*x* = 0) and Sr_2_CuWO_6_ (*x* = 1), which magnetically order at 29 and 24 K, respectively, were measured at 40 K in the short-range correlated state. Sr_2_CuTeO_6_ was also measured at 1.5, 60, and 100 K. The spin liquid-like *x* = 0.2 and 0.5 samples^47,48^ were measured at 1.5 K. The collected data were reduced using LAMP.^69^ The data were corrected for polarizer efficiency with a quartz standard and for detector efficiency with a vanadium standard. The vanadium standard was also used to normalize the data to an absolute intensity scale. The magnetic signal was isolated using *xyz* polarization analysis, which removes the nonmagnetic signal (including background).^70^ We have previously presented the raw data with limited analysis for *x* = 0.2 and 0.5 in ref (53). Magnetic diffuse scattering was fitted to an equation described later using the nonlinear curve fitting tool in OriginPro. Diffuse scattering was also modeled with a Reverse Monte Carlo (RMC) approach as implemented in SPINVERT using 8 × 8 × 6 supercells.^71^ Each SPINVERT analysis was repeated 10 times to reduce statistical noise. The experimental neutron data are available online at refs (72−74).

## 3 Results and Discussion

### 3.1 Low-Temperature Crystal Structure of *x* = 0.5

Sr_2_CuTeO_6_ and Sr_2_CuWO_6_ crystallize in the *B*-site ordered double perovskite structure with the tetragonal space group *I*4/*m*.^30,32,34,35^ The tetragonal symmetry is retained at low temperatures based on neutron diffraction measurements.^34,35^ Therefore, the square lattice of the *S* = 1/2 Cu^2+^ cations remains undistorted at low temperatures, where the quantum magnetism occurs. The d^10^/d^0^ doping does not have a significant effect on the room-temperature crystal structure, as the Sr_2_CuTe_1–*x*_W_*x*_O_6_ solid solution retains tetragonal symmetry for the full range 0 < *x* < 1.^47,48^ However, the low-temperature crystal structure has not been reported for the doped samples.

**Figure 2:**
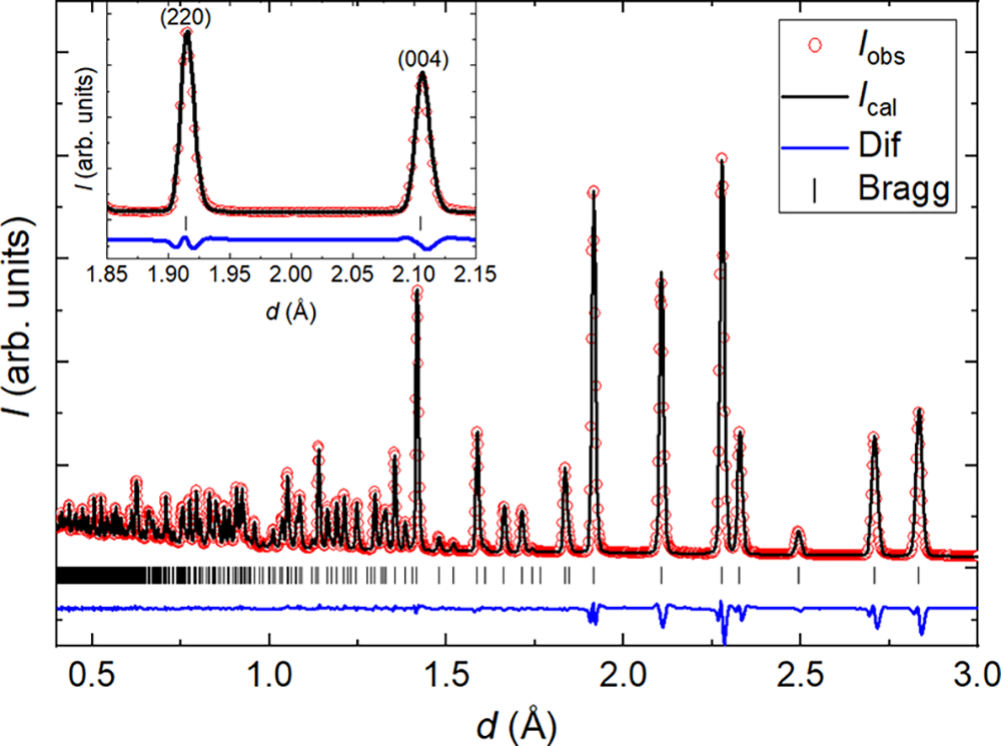
Rietveld refinement of the time-of-flight neutron diffraction data for Sr_2_CuTe_0.5_W_0.5_O_6_ (*x* = 0.5) at 5 K from bank 4 on POLARIS (2θ = 92.59°) with *R*_p_ = 2.38% and *R*_wp_ = 2.29%. The low-temperature structure remains tetragonal in the space group *I*4/*m*. Inset: close-up of the (220) and (004) reflections showing that there is no peak splitting or anisotropic line broadening, which confirms that the structure is tetragonal.

We investigated the structure of the main spin liquid composition *x* = 0.5 using neutron diffraction at 5, 100, and 300 K. The space group remains tetragonal *I*4/*m* at all temperatures, and no structural transition is observed. The refined structure at 300 K (Supporting Information) is essentially identical to the previously published structure based on laboratory X-ray diffraction.^47^Figure 2 shows the refined time-of-flight neutron data for *x* = 0.5 at 5 K. If the symmetry is lowered from tetragonal, one would expect either the (220) reflection to split or anisotropic broadening of this reflection if the splitting is too small to be resolved. The (220) and (004) reflections are highlighted in the inset. The (220) peak does not split nor is there any anisotropic line broadening. This confirms that the average structure remains tetragonal down to 5 K and, therefore, the Cu^2+^ cations are arranged in a perfect square even at low temperatures. We do not observe any superlattice reflections arising from Te/W ordering, and therefore, there is no long-range order of Te and W occupancies on the *B″*-site.

The refined crystal structure of the *x* = 0.5 sample at 5 K is presented in Table 1. The refined lattice parameters, bond distances, and angles of the *x* = 0.5 sample are similar to those of the parent phases Sr_2_CuTeO_6_ and Sr_2_CuWO_6_ due to the similar ionic radii of W^6+^ and Te^6+^.^34,35^ The *a*-parameter is mostly unaffected by doping, but the low-temperature *c* parameter decreases from 8.4521(2) Å for Sr_2_CuTeO_6_ to 8.4172(2) Å for *x* = 0.5 and, finally, 8.4125(1) Å for Sr_2_CuWO_6_. The room-temperature crystal structure of Sr_2_CuTe_1–*x*_W_*x*_O_6_ is known to follow Vegard’s law with a linear decrease in cell volume upon increasing *x*.^48^

**Table 1:** Refined Low-Temperature Crystal Structure of Sr_2_CuTe_0.5_W_0.5_O_6_ (*x* = 0.5) at 5 K Based on POLARIS Time-of-Flight Neutron Data^a^

atom	*x*	*y*	*z*	occ	*U* (Å^2^)
Sr	0	0.5	0.25	1	*U*_11_ = *U*_22_ = 0.0016(2), *U*_33_ = 0.0017(3)
Cu	0	0	0.5	1	*U*_11_ = *U*_22_ = 0.0004(3),*U*_33_ = 0.0040(5)
Te	0	0	0	0.5	*U*_11_ = *U*_22_ = 0.0013(4),*U*_33_ = 0.0012(6)
W	0	0	0	0.5	*U*_11_ = *U*_22_ = 0.0013(4),*U*_33_ = 0.0012(6)
O1	0.2015(2)	0.2917(2)	0	1	*U*_11_ = 0.0046(5), *U*_22_ = 0.0018(5), *U*_33_ = 0.0047(3),*U*_12_ = −0.0014(2)
O2	0	0	0.2267(1)	1	*U*_11_ = *U*_22_ = 0.0043(2),*U*_33_ = 0.0021(4)

a Space group *I*4/*m* with lattice parameters *a* = 5.41025(10) Å and *c* = 8.41718(18) Å. *R*_p_ = 2.60% and *R*_wp_ = 2.05% for the high-resolution bank 5 (2θ = 146.72°). The refined crystal structure is shown in Figure 1a with an origin shift of 1/2 unit cell along *c.*

The CuO_6_ octahedron is Jahn–Teller-distorted as expected with four short equatorial Cu–O1 bonds at 1.9692(11) Å and two long apical Cu–O2 bonds at 2.3004(8) Å. The Cu–O–Te/W angle is known to be more important than the in-plane Cu–O bond length for superexchange in these materials: angles closer to 180° result in stronger antiferromagnetic interactions.^30^ The Cu–O–Te/W angle increases upon doping from 158.4(2)° for Sr_2_CuTeO_6_ to 159.5(2)° for *x* = 0.5 and 160.1(2)° for Sr_2_CuWO_6_.^34,35^ The Curie–Weiss temperature of *x* = 0.5 would therefore be expected to be more negative than for Sr_2_CuTeO_6_, but the opposite is observed. This is due to the tungsten-doping (increasing *x*) quenching the nearest-neighbor *J*_1_ interaction faster than it increases the *J*_2_ interaction at low *x*,^48,52,53^ which leads to a decrease in the overall strength of antiferromagnetic interactions.

### 3.2 Pair-Distribution Function Analysis of *x* = 0.5

Our Bragg diffraction data for the *x* = 0.5 composition are well described by the average structure model with no long-range ordering on the W/Te site. However, this result does not rule out short-range ordering of W and Te. Such short-range ordering would give rise to broad (diffuse) scattering features, which are not modeled in Rietveld refinement. However, such features can be apparent at small distances *r* in the pair-distribution function (PDF), which is the Fourier transform of the diffracted intensity, appropriately normalized and corrected for background scattering.

**Figure 3:**
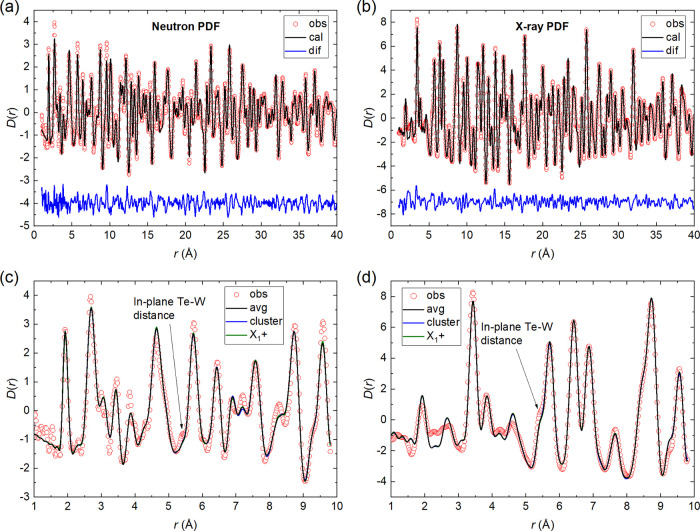
Small-box modeling of the combined neutron and synchrotron X-ray pair-distribution function data for Sr_2_CuTe_0.5_W_0.5_O_6_ (*x* = 0.5) at *T* ≈ 300 K. The wide-*r* (1 ≤ *r* ≤ 40 Å) (a) neutron and (b) X-ray PDF data are well described by the average structure. In panels (c) and (d), we fitted the neutron and X-ray PDF in the low-*r* range (1 ≤ *r* ≤ 9.8 Å) using three different models: a random distribution of Te and W (average structure), clusters of Te and W, and finally, Te–W ordering (X_1_+) on the *B″*-sites. Our experiment is not sufficient to differentiate between these models, and the fitted lines overlap. The Te–W distance shown in the figure is the lattice parameter *a* and therefore contains a contribution from Sr–Sr, Cu–Cu, and O–O partials as well.

To investigate this possibility, we analyzed our PDF data for *x =* 0.5 using Topas Academic software.^75^ Co-refinements were performed against neutron and X-ray PDF collected at *T* ≈ 300 K, as shown in Figure 3a,b. We first performed refinements of the average structure over a large *r-*range (*r*_max_ = 40 Å) to determine the instrumental parameters *Q*_damp_ and *Q*_broad_,^76^ which were fixed in subsequent refinements.

We tested three models of local Te/W occupancies against the neutron + X-ray PDF data: (i) the average-structure model, corresponding to locally random Te/W occupancies; (ii) clustering of Te and W into domains so that the measured PDF is the average of the PDFs of Sr_2_CuTeO_6_ and Sr_2_CuWO_6_ with identical lattice constants and structural parameters; (iii) a model of local anticlustering of Te and W such that W–Te neighbors are favored over W–W or Te–Te as far as possible. This last model corresponds to the X_1_^+^ irrep for Te/W ordering, and it was generated using the ISODISTORT^77^ program. For each model, the refined parameters were the scale factors for the two data sets, *a* and *c* lattice parameters, O fractional coordinates, isotropic displacement parameters for all atoms, and the low-*r* peak-sharpening function *d*_1_ defined in ref (76) (13 refined parameters for each model). Refinements were performed for each model over the low-*r* region (1.0 ≤ *r* ≤ 9.8 Å) and the wide-*r* region (1.0 ≤ *r* ≤ 40 Å).

Our neutron and X-ray PDF data and fits for the three models described above are shown in Figure 3c,d over the 1.0 ≤ *r* ≤ 9.8 Å range. Good agreement with the data is observed for all models. Unfortunately, however, the goodness of fit is not distinguishable between different models, over either the low-*r* or wide-*r* regions. That is, the PDF data are equally consistent with correlated vs random Te/W occupancies. This result is perhaps surprising, since Te and W have reasonable scattering contrast for both neutrons ((*b*_W_/*b*_Te_)^2^ = 0.70) and X-rays ((*f*_W_/*f*_Te_)^2^ = 2.03), with different contrast ratios for each experiment. However, the situation of physically different models giving rise to essentially identical PDFs is not unknown in the literature.^78^ We hypothesize that this situation is more likely in materials where the disordered atoms occupy a high-symmetry site, such as the distorted face-centered cubic lattice of We/Te atoms, where the limitations of powder data are likely to be more important.

Overall, the average-structure model yields good agreement with the measured PDF at low *r,* and there is no appreciable difference in the quality of the fit between low-*r* and wide-*r* regions. This suggests that the O1 and Cu atoms do not undergo large “size effect” displacements depending on their local Te/W coordination. In support of this conclusion, we do not observe any anomalously large displacement parameters in our Rietveld refinement (see Table 1). The displacement parameter for O1 is somewhat elongated along *c,* and the Cu displacement parameter is highly cigar-shaped yet not anomalously large, with *u*_33_(Cu) = 0.0051(5) Å^2^ at 100 K. For comparison, a well-studied quantum-spin-liquid candidate material with occupational disorder, YbMgGaO_4_, has a much larger *u*_33_(Yb) = 0.0240(4) Å^2^ at 100 K,^79^ which implies that local Cu displacements in Sr_2_CuTe_0.5_W_0.5_O_6_ are small compared to local Yb displacements in YbMgGaO_4_. We also refined the anisotropic displacements for Sr_2_CuWO_6_ based on published time-of-flight neutron data^32^ (see the Supporting Information). The Cu displacement in Sr_2_CuWO_6_ is similarly anisotropic with *u*_33_(Cu) = 0.0045(3) Å^2^ at 100 K. As such, the anisotropy is likely to be related to phonons and the effect of disorder is more subtle. The observation of minimal local distortions in Sr_2_CuTe_0.5_W_0.5_O_6_ is consistent with the fact that Sr_2_CuTeO_6_ and Sr_2_CuWO_6_ have nearly identical crystal structures.

### 3.3 Magnetic Order in the W-Rich Materials (*x* = 0.9, 0.8, and 0.7)

One of the main reasons the Sr_2_CuTe_1–*x*_W_*x*_O_6_ system is so interesting is the fact that the parent phases Sr_2_CuTeO_6_ (*x* = 0) and Sr_2_CuWO_6_ (*x* = 1) have different magnetic structures: *x* = 0 has the Néel structure with **k** = (1/2, 1/2, 0) and *x* = 1 has the columnar structure with **k** = (0, 1/2, 1/2).^34,35^ The compositions *x* = 0.9, 0.8, and 0.7 are also known to magnetically order at *T*_N_ = 15, 11, and 7 K based on muon measurements.^48^ The magnetic structure for these compositions was proposed to be columnar,^48^ which was supported by later neutron diffraction experiments revealing the presence of the (0 1/2 1/2) reflection around |*Q*| = 0.68 Å^–1^.^51^ However, diffraction data were only presented for this one reflection. We have reinvestigated the type of magnetic order in *x* = 0.9, 0.8, and 0.7 using high-intensity neutron diffraction.

**Figure 4:**
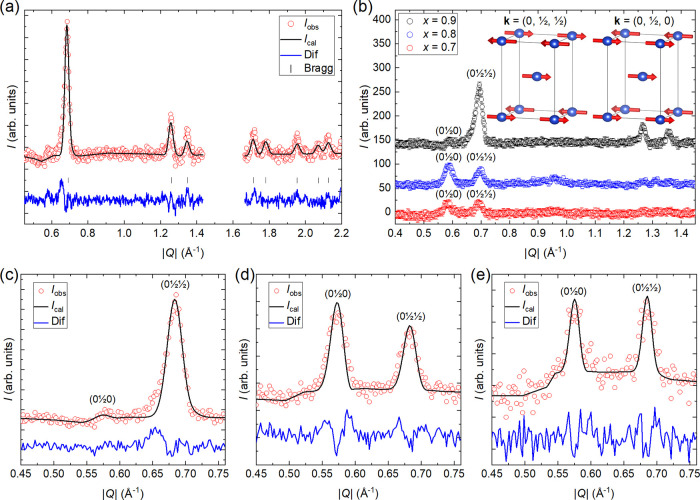
(a) Rietveld refinement of the magnetic neutron diffraction data for Sr_2_CuTe_0.1_W_0.9_O_6_ (*x* = 0.9) at 2 K obtained by subtracting the nuclear scattering measured at 30 K. The propagation vector is **k** = (0, 1/2, 1/2) with a refined moment of 0.45(1) μ_B_ along the *a* direction (*R*_mag_ = 13.3%). (b) Comparison of the magnetic neutron diffraction data for Sr_2_CuTe_1–*x*_W_*x*_O_6_ samples (*x* = 0.9, 0.8, and 0.7) at 2 K. Magnetic scattering has been normalized using the nuclear scale factor to account for differences in sample masses and counting times. A number of magnetic Bragg peaks are observed for *x* = 0.9 with the main peak being (0 1/2 1/2). For *x* = 0.8 and 0.7, the intensity of the magnetic reflections is significantly reduced. The (0 1/2 1/2) peak is retained for these compositions, but a new peak at (0 1/2 0) is observed. Panel (b) inset: magnetic structure of *x* = 1 and 0.9 with propagation vector **k** = (0, 1/2, 1/2) and the proposed magnetic structure for the second magnetic phase in *x* = 0.8 and 0.7 with propagation vector **k** = (0, 1/2, 0). The ordering along *c* changes from antiferromagnetic to ferromagnetic. Bottom panels: two-phase magnetic refinement of Sr_2_CuTe_1–*x*_W_*x*_O_6_ samples: (c) *x* = 0.9, (d) *x* = 0.8, and (e) *x* = 0.7. The *x* = 0.9 sample is almost entirely in the **k** = (0, 1/2, 1/2) magnetic structure that is also observed in *x* = 1. For *x* = 0.8, we obtain phase fractions of 65(5)% **k** = (0, 1/2, 0) and 35(5)% **k** = (0, 1/2, 1/2), and for *x* = 0.7, we find 55(8)% **k** = (0, 1/2, 0) and 45(8)% **k** = (0, 1/2, 1/2).

Refined magnetic Bragg scattering for *x* = 0.9 is shown in Figure 4a. Clear magnetic Bragg peaks are observed at positions corresponding to the propagation vector **k** = (0, 1/2, 1/2). Magnetic scattering is almost identical to previous reports on *x* = 1.^35^ The propagation vector has only one irreducible representation Γ_2_, which has three basis vectors along *a*, *b*, and *c*. Similar to *x* = 1, setting the moment along *a* resulted in the best fit. The refined Cu^2+^ ordered moment was found to be 0.45(1) μ_B_, which is slightly lower than 0.57(1) μ_B_ reported for *x* = 1.^35^ This magnetic structure is shown in the inset in Figure 4b. A comparison of magnetic Bragg scattering for *x* = 0.9, 0.8, and 0.7 is presented in panel (b). The main peak at |*Q*| = 0.68 Å corresponding to (0 1/2 1/2) is still observed in *x* = 0.8 and 0.7, but magnetic scattering is significantly weaker and the other reflections corresponding to **k** = (0, 1/2, 1/2) can no longer be resolved. Surprisingly, we observe significant magnetic scattering at |*Q*| = 0.58 Å^–1^ corresponding to (0 1/2 0). This reflection is not allowed for the propagation vector **k** = (0, 1/2, 1/2), and therefore, this reflection must belong to another propagation vector. Very weak scattering at this position also occurs in the *x* = 0.9 sample.

The magnetic propagation vectors of materials often correspond to high-symmetry points of the Brillouin zone, and therefore, these are an excellent starting point for searching for reasonable **k**-vectors. The columnar magnetic structure of *x* = 1 and 0.9 with **k** = (0, 1/2, 1/2) corresponds to the high-symmetry point X. While one might expect **k** = (0, 1/2, 0) observed in *x* = 0.8 and 0.7 to also be a high-symmetry point of the Brillouin zone, it is not one in the space group *I*4/*m*. This is due to the *I*-centering of the lattice and the relationship between the conventional unit cell and the primitive cell. As a consequence, this magnetic structure belongs to the line SM with **k** = (0, *a*, 0) and *a* = 0.500(2).

Symmetry-allowed magnetic structures for **k** = (0, 1/2, 0) were evaluated using BASIREPS.^60^ Two irreducible representations were found: Γ_mag_ = Γ_1_ + Γ_2_. Γ_1_ corresponds to magnetic moments along *c*, while Γ_2_ corresponds to moments within the *ab* plane. Given that we were only able to resolve the main magnetic peak, we are unable to determine the moment directions. Our proposed magnetic structure for *x* = 0.8 and 0.7 with **k** = (0, 1/2, 0) is presented in the inset in Figure 4b with magnetic moments along *a*. This structure corresponds to columnar antiferromagnetic order in the square-layers in the *ab* plane similar to *x* = 1. However, the interlayer coupling along *c* is now ferromagnetic instead of antiferromagnetic.

The presence of the forbidden (0 1/2 0) reflection in the magnetic scattering of Sr_2_CuTe_1–*x*_W_*x*_O_6_ samples (*x* = 0.8 and 0.7) can be interpreted in two ways. It can be due to magnetic phase separation such that parts of the sample have **k** = (0, 1/2, 1/2) and parts have the **k** = (0, 1/2, 0) magnetic structure. The other possibility is that there is a complex multi-**k** structure that accounts for all the observed magnetic scattering. Weak magnetic scattering makes this distinction complicated, since we can only observe two magnetic Bragg peaks. In the case of magnetic phase separation, we would expect the intensity of the two observed magnetic peaks to vary freely between samples. If the materials have a multi-**k** structure, the relative intensities should remain the same and be an integer ratio.

We investigated this by integrating over the (0 1/2 0) and (0 1/2 1/2) reflections. The intensity ratios *A*_(0 1/2 0)_/*A*_(0 1/2 1/2)_ were found to be 0.07 for *x* = 0.9, 1.36 for *x* = 0.8, and 1.13 for *x* = 0.7. The intensity ratio of the (0 1/2 0) and (0 1/2 1/2) peaks changes with *x*, which suggests magnetic phase separation rather than a multi-**k** structure. To evaluate the magnetic phase fractions in the samples, two-phase magnetic refinement of the main magnetic peaks was carried out using FULLPROF (see Figure 4c–e). The *x* = 0.9 sample is almost entirely in the **k** = (0, 1/2, 1/2) magnetic structure shared by the parent phase Sr_2_CuWO_6_ (*x* = 1) with only 5(3)% of **k** = (0, 1/2, 0). For *x* = 0.8, the phase fractions are 35(5)% **k** = (0, 1/2, 1/2) and 65(5)% **k** = (0, 1/2, 0) with an ordered moment of 0.34(1) μ_B_. Magnetic scattering in *x* = 0.7 is weak, leading to higher uncertainties of 45(8)% **k** = (0, 1/2, 1/2) and 55(8)% **k** = (0, 1/2, 0) and an ordered moment of only 0.24(1) μ_B_.

The widths of the magnetic Bragg peaks are wider than the nuclear peaks in these materials. For *x* = 0.9, we obtain fwhm’s of 0.64(2)° for the main magnetic peak (0 1/2 1/2) and 0.46(1)° for the first nuclear peak (011). The fwhm’s for *x* = 0.8 are 0.69(3)° and 0.68(5)° for the (0 1/2 0) and (0 1/2 1/2) magnetic peaks and 0.47(1)° for (011), and for *x* = 0.7, we obtain 0.51(8)°, 0.55(6)°, and 0.47(1)°, respectively. The nuclear peak widths are dominated by instrumental broadening as opposed to sample broadening. This supports the presence of size broadening for the magnetic peaks: the size of the magnetic domains is smaller than the crystallite size. The crystallite sizes for all samples are on the order of ∼1 μm.^48^ Estimation of the sizes of the magnetic domains is complicated by the significant instrumental line broadening on D20. While taking this broadening into account, we estimate the apparent size of the magnetic domains in *x* = 0.9 to be 47(1) nm from the Scherrer equation using integral breadths β obtained from Thompson–Cox–Hastings profile parameters.^80,81^ Clustering into Te-rich and W-rich regions could lead to the presence of both magnetic structures within a single crystallite. Unfortunately, we were unable to determine the presence or absence of clustering in the X-ray PDF analysis.

The magnetic interactions in Sr_2_CuTe_1–*x*_W_*x*_O_6_ materials are highly two-dimensional. The interlayer interaction *J*_c_ is an extended Cu–O–W/Te–O–Cu superexchange similar to *J*_2_ but much weaker due to the fully occupied Cu dz^2^ orbitals. In Sr_2_CuWO_6_ (*x* = 1), the interlayer exchange is antiferromagnetic with *J*_c_ = −0.01 meV, while the dominant in-plane exchange is *J*_2_ = −9.5 meV. As a result of the weak *J*_c_, inelastic scattering in the forbidden (0 1/2 0) position is observed even in undoped Sr_2_CuWO_6_.^27^ The *J*_c_ interaction is ferromagnetic in Sr_2_CuTeO_6_ (*x* = 0) following the trend observed in the sign of *J*_2_. As a result, it appears that Te-doping (decreasing *x*) disrupts the interlayer interactions, leading to the appearance of the competing **k** = (0, 1/2, 0) structure. Similar magnetic phase separation into structures with different interlayer ordering is also observed in the solid solution Sr_2_Cr_1.85_Mn_1.15_As_2_O_2_, which has a square layer of Cr^2+^ (*S* = 2) cations.^82^ The weak interlayer interaction is ultimately responsible for magnetic ordering in the Sr_2_CuTe_1–*x*_W_*x*_O_6_ materials, since magnetic order in two dimensions at nonzero temperature is forbidden by the Mermin–Wagner theorem when the interactions are isotropic.^83^ As such, the disorder in *J*_c_ could be the cause of the quantum phase transition from columnar magnetic order in the W-rich side to the spin liquid-like state at *x* ≈ 0.6. This is supported by our diffuse magnetic scattering analysis on *x* = 0.5 and *x* = 0.2 samples described later, which shows that the average interlayer spin correlations are weak and antiferromagnetic in *x* = 0.5 but change to weakly ferromagnetic for *x* = 0.2.

### 3.4 Diffuse Magnetic Scattering in the Spin Liquid-like Materials (*x* = 0.2 and 0.5)

The Sr_2_CuTe_1–*x*_W_*x*_O_6_ parent phases *x* = 0 (Sr_2_CuTeO_6_) and *x* = 1 (Sr_2_CuWO_6_) are known to have short-range correlated magnetic states above *T*_N_ based on inelastic neutron scattering studies.^26,27^ Spin correlations persist up to at least 2*T*_N_ ≈ 60 K in both compounds. We previously proposed that the spin liquid-like state in *x* = 0.5 could be related to the columnar-type short-range correlated state in *x* = 1 based on inelastic neutron scattering data.^52^ Similarly, the spin liquid-like state between *x* = 0.05 and 0.2 has been proposed to have Néel-type short-range correlations.^51^ We can test this hypothesis by measuring diffuse magnetic scattering in the spin liquid-like phases and in the short-range correlated states of the parent phases. This allows us to model the local spin correlations in these materials for the first time.

**Figure 5:**
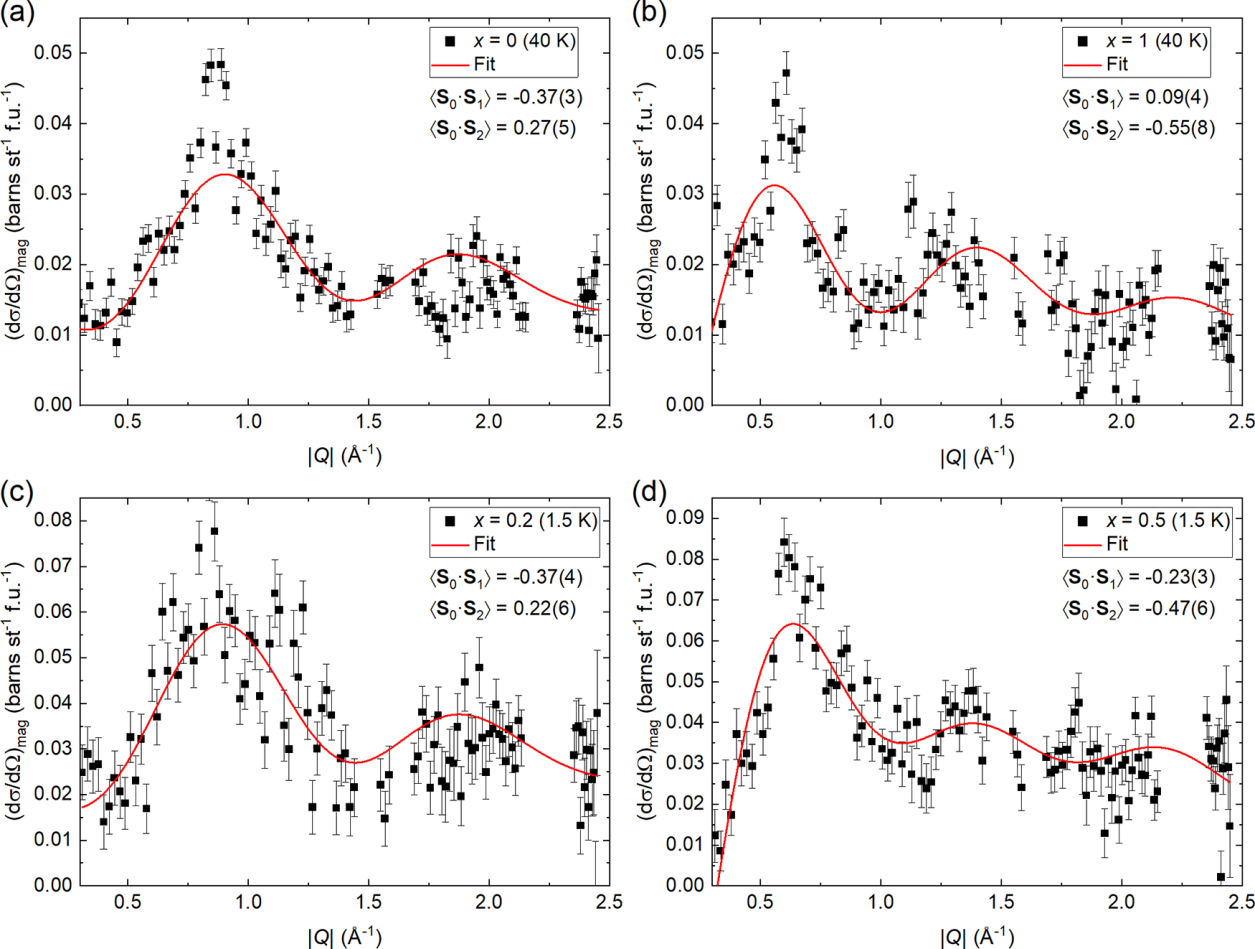
Magnetic diffuse scattering of the Sr_2_CuTe_1–*x*_W_*x*_O_6_ parent phases (a) *x* = 0 (Sr_2_CuTeO_6_) and (b) *x* = 1 (Sr_2_CuWO_6_) at 40 K in the short-range correlated state above *T*_N_ and the spin-liquid-like phases (c) *x* = 0.2 and (d) *x* = 0.5 at 1.5 K. The red lines are fits to eq 1. Magnetic diffuse scattering has a peak at |*Q*| ≈ 0.85 Å^–1^ for *x* = 0 and at |*Q*| ≈ 0.6 Å^–1^ for *x* = 1. These are related to the Néel and columnar magnetic ordering below *T*_N_, respectively. The *x* = 0.2 data have a peak at |*Q*| ≈ 0.85 Å^–1^ similar to *x* = 0 above *T*_N_. For *x* = 0.5, the peak is observed at |*Q*| ≈ 0.6 Å^–1^ similar to *x* = 1 above *T*_N_. Diffuse magnetic scattering in the two spin liquid-like samples *x* = 0.2 and *x* = 0.5 is clearly different but also clearly related to the two parent phases above *T*_N_.

The diffuse magnetic scattering of Sr_2_CuTe_1–*x*_W_*x*_O_6_ samples extracted from our D7 experiment is shown in Figure 5. The parent phases *x* = 0 (a) and *x* = 1 (b) were measured above *T*_N_ at 40 K, where they are in a short-range correlated magnetic state. The other samples measured were *x* = 0.2 (c) and *x* = 0.5 (d) at 1.5 K, which are in the spin liquid-like region and lack long-range magnetic order. Diffuse scattering for *x* = 0 at 40 K has a peak around |*Q*| ≈ 0.85 Å^–1^. This is related to the Néel magnetic order at low temperatures with the Bragg reflection (1/2 1/2 0) at |*Q*| = 0.82 Å^–1^.^34^ Diffuse scattering for *x* = 1 at 40 K is very different, with a main peak around |*Q*| ≈ 0.6 Å^–1^. This is related to the columnar magnetic order below *T*_N_, which has a magnetic Bragg reflection (0 1/2 1/2) at |*Q*| = 0.68 Å^–1^ and inelastic scattering at the forbidden (0 1/2 0) position at |*Q*| = 0.58 Å^–1^.^27^ The scattering from the *x* = 0.2 sample at 1.5 K is similar to *x* = 0, although the peak at |*Q*| ≈ 0.85 Å^–1^ is significantly broader in *x* = 0.2 with fwhm’s of 0.27(8) and 0.61(15) Å^–1^, respectively. This supports the hypothesis that spin correlations in *x* = 0.2 are Néel-like. Diffuse scattering for *x* = 0.5 is similar to *x* = 1 with a peak around |*Q*| ≈ 0.6 Å^–1^. This supports the hypothesis that spin correlations in *x* = 0.5 are columnar-like.

The low incident energy of *E*_i_ = 3.55 meV is a limitation in our D7 experiment. Ideally, the incident energy would be high enough to capture all features of the inelastic neutron scattering spectrum.^84^ In the case of Sr_2_CuTe_1–*x*_W_*x*_O_6_, significant inelastic scattering is observed up to at least 15 meV.^26,27,53^ Fitting the high-temperature (100 K) diffuse scattering of *x* = 0 to the Cu^2+^ magnetic form factor results in μ_eff_^2^ = 0.91(2) μ_B_^2^, which is only one-third of the expected μ_eff_^2^ = 3 μ_B_^2^ for a *S* = 1/2 cation. We can estimate the effect of the missing magnetic scattering by integrating inelastic neutron scattering data up to higher energies. These cuts for *x* = 0.2 and 0.5 (Supporting Information) show that our D7 experiment does capture the essential features of diffuse scattering.

To quantify the spin correlations in Sr_2_CuTe_1–*x*_W_*x*_O_6_, the observed magnetic scattering cross sections were fitted to^85^
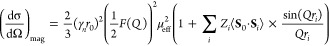
1

where *F*(*Q*) is the magnetic form factor of Cu^2+^, μ_eff_^2^ = *g*^2^*S*(*S* + 1), *Z*_*i*_ is the number of neighboring spins at distance *r*_*i*_, and ⟨**S**_0_·**S**_*i*_⟩ is the average spin correlation between a central spin and its neighbors at distance *r*_*i*_. The spin correlations have been normalized such that ⟨**S**_0_·**S**_*i*_⟩ = 1 (−1) corresponds to complete (anti)ferromagnetic alignment of spins. We considered only the nearest-neighbor spins at *r*_1_ ≈ 5.4 Å and the in-plane next-nearest-neighbor spins at *r*_2_ ≈ 7.6 Å. These correspond to the spins along the side (*J*_1_ interaction) and diagonal (*J*_2_ interaction) of the square, respectively. Thus, we have three fitting parameters: μ_eff_^2^ and the spin correlations ⟨**S**_0_·**S**_1_⟩ and ⟨**S**_0_·**S**_2_⟩. For complete Néel antiferromagnetic order, ⟨**S**_0_·**S**_1_⟩ = −1 and ⟨**S**_0_·**S**_2_⟩ = 1. For columnar order, ⟨**S**_0_·**S**_1_⟩ = 0 and ⟨**S**_0_·**S**_2_⟩ = −1.

The fits to eq 1 are shown in Figure 5. The fitting captures the main features of the scattering for all samples, but the model is missing significant intensity at the main peak positions at |*Q*| ≈ 0.85 Å^–1^ (*x* = 0) and |*Q*| ≈ 0.6 Å^–1^ (*x* = 0.5 and 1). Magnetic diffuse scattering becomes narrower and more Bragg-like when the temperature approaches *T*_N_, which is not captured by this model. This could explain why the main peak intensity does not fit well for *x* = 0 and *x* = 1. It should be noted that eq 1 is a simple model including only the two nearest-neighbor in-plane correlations, which are assumed to be fully independent of each other, and Heisenberg spins.^71^ Moreover, our experiment does not capture the full spectral weight, which makes the fitting more difficult. There is an additional feature at |*Q*| ≈ 1.13 Å in the *x* = 0.2 data that is not captured by the model. We are not sure whether this feature is real or an effect of statistics and binning. This feature is not present in the integrated inelastic neutron scattering data (Supporting Information) for the same sample.

**Figure 6:**
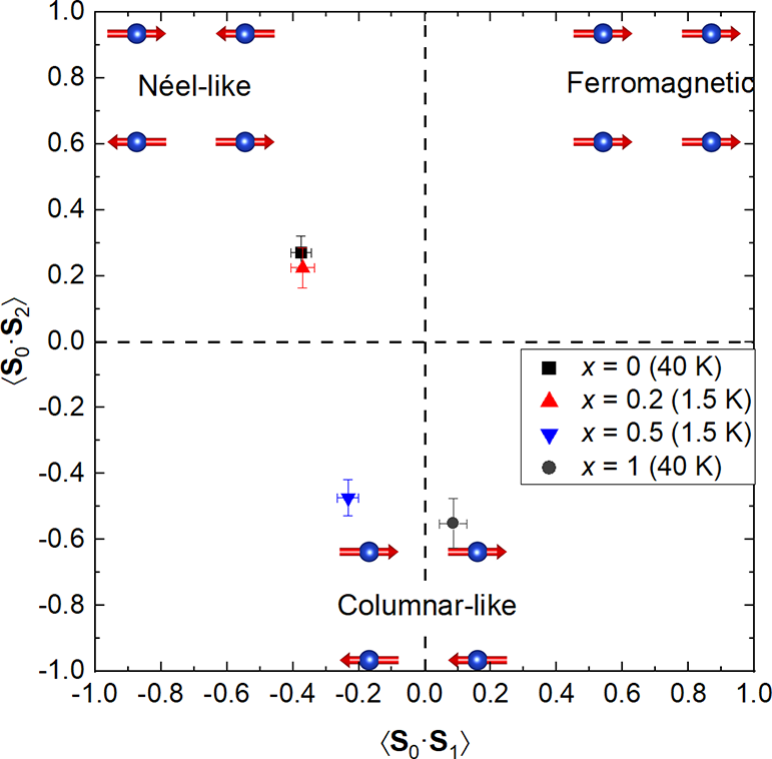
Spin correlations in Sr_2_CuTe_1–*x*_W_*x*_O_6_ obtained from fitting magnetic diffuse scattering to eq 1. The spin correlations in *x* = 0 above *T*_N_ are strongly Néel-like with antiferromagnetic ⟨**S**_0_·**S**_1_⟩ and ferromagnetic ⟨**S**_0_·**S**_2_⟩. The spin correlations in the spin liquid-like state in *x* = 0.2 are Néel-like and very similar to *x* = 0. However, the spin liquid-like state in *x* = 0.5 has very different correlations: weak antiferromagnetic ⟨**S**_0_·**S**_1_⟩ and strong antiferromagnetic ⟨**S**_0_·**S**_2_⟩. The latter is a feature of columnar-like spin correlations. Finally, for *x* = 1 above *T*_N_, we obtain ⟨**S**_0_·**S**_1_⟩ ≈ 0 and strongly antiferromagnetic ⟨**S**_0_·**S**_2_⟩ as expected of columnar-like spin correlations.

The obtained spin correlations are plotted in Figure 6. For *x* = 0 at 40 K, we obtain ⟨**S**_0_·**S**_1_⟩ = −0.37(3) and ⟨**S**_0_·**S**_2_⟩ = 0.27(5). These can be characterized as Néel-type correlations linked to the Néel magnetic order in *x* = 0 at low temperatures, where ⟨**S**_0_·**S**_1_⟩ = −1 and ⟨**S**_0_·**S**_2_⟩ = 1. The spin correlations in *x* = 0.2 at 1.5 K are very similar to *x* = 0 at 40 K with ⟨**S**_0_·**S**_1_⟩ = −0.37(4) and ⟨**S**_0_·**S**_2_⟩ = 0.22(6) and are therefore Néel-like. A significant change in the spin correlations occurs at *x* = 0.5, which is clearly different from *x* = 0 and 0.2. For *x* = 0.5 at 1.5 K, we obtain ⟨**S**_0_·**S**_1_⟩ = −0.23(3) and ⟨**S**_0_·**S**_2_⟩ = −0.47(6). The correlations are now mainly columnar-like with some Néel-like nearest-neighbor correlations remaining. The spin correlations in *x* = 1 at 40 K are entirely columnar-like with ⟨**S**_0_·**S**_1_⟩ = 0.09(4) and ⟨**S**_0_·**S**_2_⟩ = −0.55(8), consistent with the expected ⟨**S**_0_·**S**_1_⟩ = 0 and ⟨**S**_0_·**S**_2_⟩ = −1 for complete columnar ordering.

**Figure 7:**
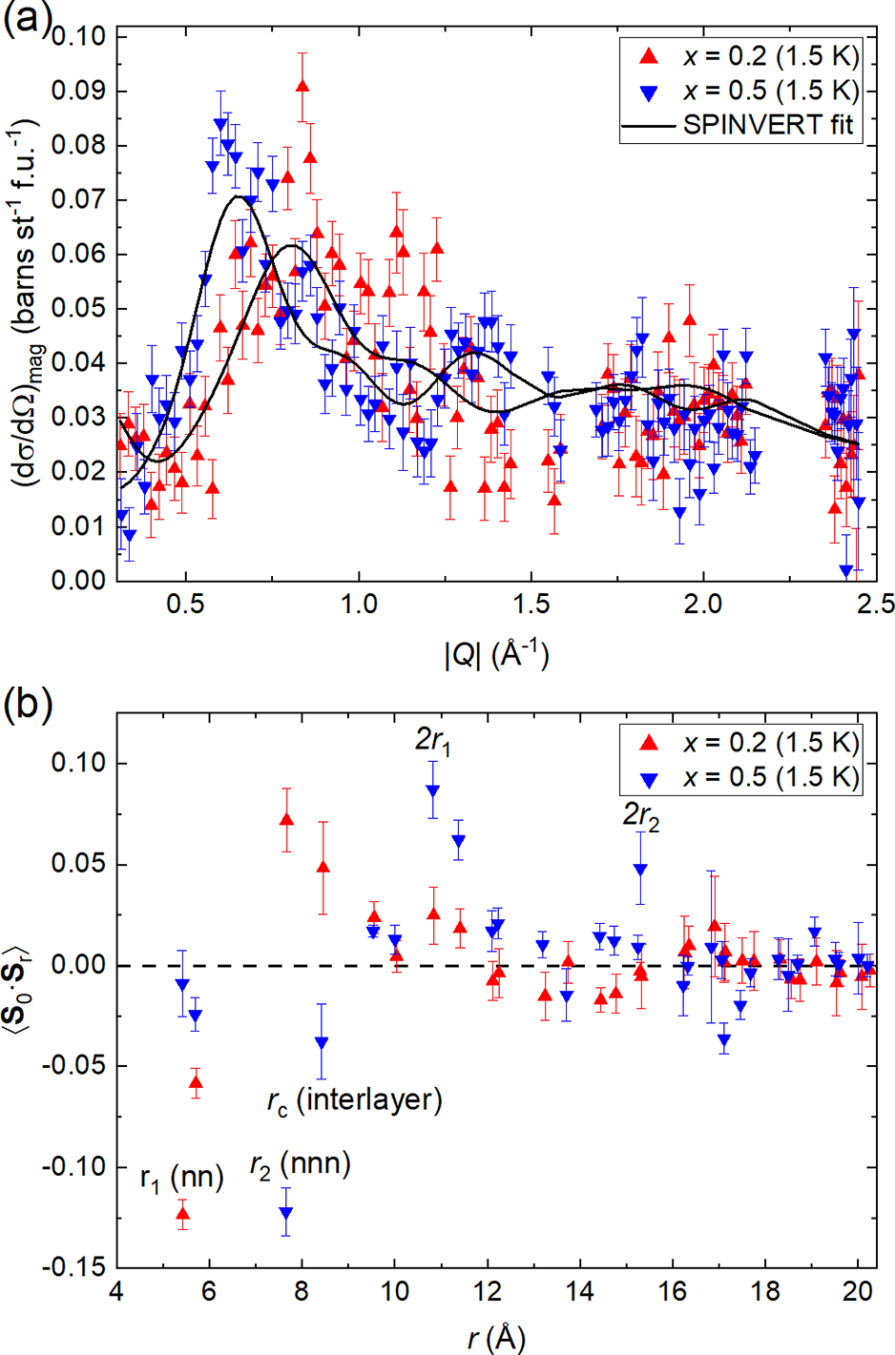
(a) SPINVERT fits to diffuse magnetic scattering of *x* = 0.2 and 0.5 samples at 1.5 K. The quality of the fits is limited by weak magnetic scattering and limited energy coverage. The maximum in scattering is observed at different |*Q*| positions in the two samples: at |*Q*| ≈ 0.85 Å^–1^ in *x* = 0.2 and at |*Q*| ≈ 0.6 Å^–1^ in *x* = 0.5. This indicates a significant difference in the spin correlations of these two materials. There is a feature in the *x* = 0.2 data at |*Q*| ≈ 1.13 Å^–1^ that is not captured in the model. (b) Radial spin correlation functions of the *x* = 0.2 and 0.5 samples obtained from the SPINVERT fits. Spin correlations in the *x* = 0.2 are Néel-type with strong antiferromagnetic ⟨**S**_0_·**S**_1_⟩ and ferromagnetic ⟨**S**_0_·**S**_2_⟩. The correlations are columnar-type in *x* = 0.5 with negligible ⟨**S**_0_·**S**_1_⟩ and strong antiferromagnetic ⟨**S**_0_·**S**_2_⟩ as expected. The interlayer spin correlation is ferromagnetic in *x* = 0.2 and antiferromagnetic in *x* = 0.5.

For comparison, we also fitted diffuse magnetic scattering using a reverse Monte Carlo (RMC) method as implemented in SPINVERT.^71^ These fits are shown in Figure 7 for *x* = 0.2 and *x* = 0.5 and in the Supporting Information for Sr_2_CuTeO_6_ and Sr_2_CuWO_6_. The main spin correlations obtained using SPINVERT broadly support our fitting results with the exception of *x* = 0.5, where the weaker ⟨**S**_0_·**S**_1_⟩ is moderately antiferromagnetic in the eq 1 fit but zero within the experimental error (as expected) in the SPINVERT fit. The same main conclusion of Néel-like correlations in *x* = 0.2 and columnar-like correlations in *x* = 0.5 is observed using both fitting methods.

One advantage of the RMC approach is that spin correlations up to further neighbors can easily be evaluated. The obtained spin correlations for *x* = 0.2 are Néel-type: strong antiferromagnetic nearest-neighbor ⟨**S**_0_·**S**_1_⟩ correlations (*r*_1_ ≈ 5.4 Å) and ferromagnetic in-plane next-nearest-neighbor correlations ⟨**S**_0_·**S**_2_⟩ (*r*_2_ ≈ 7.6 Å). The spin correlations at distance 2*r*_1_ are weakly ferromagnetic, whereas at 2*r*_2_, they are nearly zero. The first three in-plane spin correlations are as expected for Néel-type correlations. The spin correlations of *x* = 0.2 at 1.5 K are very similar to those of Sr_2_CuTeO_6_ at 40 K. For the *x* = 0.5 sample, we obtain ⟨**S**_0_·**S**_1_⟩ ≈ 0 and strong antiferromagnetic ⟨**S**_0_·**S**_2_⟩ correlations. The spin correlations at 2*r*_1_ and 2*r*_2_ are ferromagnetic. All four main in-plane spin correlations in *x* = 0.5 are precisely as expected for columnar-type correlations. This would suggest that the spin correlations in *x* = 0.5 are purely of columnar type without any nearest-neighbor Néel-type contribution. Moreover, the in-plane spin correlations in *x* = 0.5 are very similar to those of Sr_2_CuWO_6_ above *T*_N_ (see the Supporting Information). A comparison of the obtained and expected spin correlations is provided in the Supporting Information.

The interlayer spin correlations ⟨**S**_0_·**S**_*c*_⟩ at *r*_c_ ≈ 8.4 Å can also be evaluated using SPINVERT. These are of interest since the magnetic phase separation observed in the W-rich samples results in different magnetic ordering between the layers. The interlayer spin correlations are ferromagnetic in Sr_2_CuTeO_6_ and antiferromagnetic in Sr_2_CuWO_6_ at 40 K, which is consistent with their magnetic structures below *T*_N_.^34,35^ The interlayer spin correlation in *x* = 0.2 at 1.5 K is also ferromagnetic but weaker than in Sr_2_CuTeO_6_ at 40 K. For *x* = 0.5, we obtained an antiferromagnetic interlayer spin correlation, which was similarly weaker than in Sr_2_CuWO_6_ at 40 K. These results show that the interlayer spin correlations become weaker and change sign in the doped samples, which along with our neutron diffraction results supports disorder in the interlayer magnetic interactions as the reason for suppression of magnetic order at *x* ≈ 0.6.

To summarize, the spin liquid-like states in Sr_2_CuTe_1–*x*_W_*x*_O_6_ with *x* = 0.2 and *x* = 0.5 have short-range spin correlations very similar to the parent phases *x* = 0 and *x* = 1, respectively, above *T*_N_. The spin correlations in *x* = 0 and 0.2 are Néel-like, while the correlations in *x* = 0.5 and 1 are mainly columnar-like. The crossover from Néel to columnar correlations occurs somewhere between *x* = 0.2 and 0.4, because the inelastic neutron scattering data for *x* = 0.4 and 0.5 are very similar.^53^ It is unclear whether *x* = 0.2 and *x* = 0.5 have the same ground state given the significant differences in spin correlations. Significant Néel-type correlations are expected in the random-singlet state^55^ as we observe for *x* = 0.2. The presence of Néel-type correlations in *x* = 0.5 is less certain. It is clear that the main spin correlations are columnar-type in *x* = 0.5, but some weak Néel-like correlations were found in the fits to eq 1. However, we did not observe these correlations in the SPINVERT analysis of the same data. As such, our experiment suggests that *x* = 0.2 and *x* = 0.5 have a different ground state and that there is a quantum critical point between them. This could be a quantum critical point between two different types of random-singlet states or a random-singlet state and a state with weakly frozen moments.^56^ We expect this quantum critical point, should it exist, to occur between 0.2 < *x* < 0.4, where the spin correlations change from Néel to columnar. This could be further investigated by muon spectroscopy as the scaling behavior of the muon spin relaxation rate should change in the presence of a critical point.^51^

## 4 Conclusions

We have investigated different compositions of the *S* = 1/2 square-lattice antiferromagnet Sr_2_CuTe_1–*x*_W_*x*_O_6_ using neutron and X-ray scattering. The average crystal structure of the spin-liquid-like *x* = 0.5 sample remains tetragonal down to 5 K confirming a square magnetic lattice. Our PDF analysis showed the local structure is overall well described by the average structure, although we were unable to distinguish between different models of Te and W correlations (random, clustering or ordering). Our neutron diffraction experiments of the W-rich magnetically ordered materials reveal the presence of columnar magnetic order for *x* = 0.7–1. Surprisingly, magnetic phase separation was observed for *x* = 0.7 and 0.8 with part of the sample ordering ferromagnetically and part antiferromagnetically along *c*, while the columnar order in the *ab*-plane was preserved. This shows replacing W with Te leads to disorder in the interlayer interactions, which could be the origin of the transition to the spin-liquid-like state at *x* ≈ 0.6.

The spin correlations of the spin-liquid-like phases *x* = 0.2 and 0.5 were investigated using polarized neutrons. The spin-correlations in *x* = 0.2 are Néel-like, and very similar to the short-range correlated state observed above *T*_N_ in Sr_2_CuTeO_6_. The spin correlations in *x* = 0.5 are mainly columnar-type with potentially some weak Néel-like correlations remaining, and similar to the short-range correlated state above *T*_N_ in Sr_2_CuWO_6_. Despite both compositions being in the spin-liquid-like region, the spin correlations are very different. This suggests the ground states are also different, because Néel-type correlations are expected in the random-singlet state. If so, a quantum critical point would be expected between 0.2 < *x* < 0.4, where the spin correlations change from Néel to columnar-like.

Our results highlight the complexity of Sr_2_CuTe_1–*x*_W_*x*_O_6_ as a *S* = 1/2 square-lattice antiferromagnet with frustration and disorder in the local in-plane and interlayer interactions. The strong suppression of Néel order at *x* ≈ 0.05 and the related quantum critical point has previously garnered significant attention.^50,51,55^ Our results show that further investigation is warranted at higher doping levels both at the other known quantum critical point at *x* ≈ 0.6 and our proposed new quantum critical point between 0.2 < *x* < 0.4. Given that the d^10^/d^0^ substitution approach for tuning magnetism is applicable to many 3d transition metal oxides,^37^ our insights have relevance to other systems such as SrLaSb_1–*x*_Nb_*x*_CuO_6_ or the spin ladder Ba_2_CuTe_1–*x*_W_*x*_O_6_.^40,46^

## Supporting Information Available

Crystal structure of Sr_2_CuTe_0.5_W_0.5_O_6_, refinement of anisotropic *U* parameters for Sr_2_CuWO_6_, magnetic scattering in the ordered W-rich phases, magnetic scattering of Sr_2_CuTeO_6_ at various temperatures, integrated inelastic neutron scattering data, and SPINVERT fits of diffuse magnetic scattering (PDF)
CIF file for Sr_2_CuTe_0.5_W_0.5_O_6_ at 5 K (CIF)
CIF file for Sr_2_CuTe_0.5_W_0.5_O_6_ at 300 K (CIF)

## Supplementary Material

cm3c02535_si_001.pdf

cm3c02535_si_002.cif

cm3c02535_si_003.cif

## ABBREVIATIONS

PDF: pair distribution function
RMC: reverse Monte Carlo
fwhm: full width at half-maximum
