# Supplementary material for: Structure, Spin Correlations, and Magnetism of the S = 1/2 Square-Lattice Antiferromagnet Sr2CuTe1–xWxO6 (0 ≤ x ≤ 1)
Source: Chem Mater. 2023 Dec 25;36(1):501–13. doi: 10.1021/acs.chemmater.3c02535 (PMC10782448; doi:10.1021/acs.chemmater.3c02535)
Supplement: Supplementary file 1 — cm3c02535_si_001.pdf [file cm3c02535_si_001.pdf]

Supporting information

Structure, spin correlations and magnetism of the *S* = 1/2 square-lattice antiferromagnet $Sr_2CuTe_{1-x}W_xO_6$ ($0 \leq x \leq 1$)

Otto H. J. Mustonen,[1,2]* Ellen Fogh,[3]* Joseph A. M. Paddison,[4] Lucile Mangin-Thro,[5] Thomas Hansen,[5] Helen Y. Playford,[6] Maria Diaz-Lopez,[7] Peter Babkevich,[3] Sami Vasala,[8] Maarit Karppinen,[9] Edmund J. Cussen,[2] Henrik M. Rønnow,[3] Helen C. Walker[6]*

[1] School of Chemistry, University of Birmingham, Birmingham B15 2TT, United Kingdom
[2] Department of Material Science and Engineering, University of Sheffield, Sheffield S1 3JD, United Kingdom
[3] Laboratory for Quantum Magnetism, Institute of Physics, École Polytechnique Fédérale de Lausanne (EPFL), CH-1015 Lausanne, Switzerland
[4] Materials Science and Technology Division, Oak Ridge National Laboratory, Oak Ridge, Tennessee 37831, USA
[5] Institut Laue Langevin, 71 Avenue des Martyrs, CS 20156, F-38042 Grenoble Cedex 9, France
[6] ISIS Neutron and Muon Source, Rutherford Appleton Laboratory, Chilton, Didcot OX11 OQX, United Kingdom
[7] Université Grenoble Alpes, CNRS, Grenoble INP, Institut Néel, 38000 Grenoble, France
[8] ESRF - The European Synchrotron, 38000 Grenoble, France
[9] Department of Chemistry and Materials Science, Aalto University, FI-00076, Espoo, Finland

Table of contents

Crystal structure of $Sr_2CuTe_{0.5}W_{0.5}O_6$ (x = 0.5) 2
Refinement of anisotropic *U* parameters for Sr_2CuWO_6 5
Magnetic scattering in the ordered W-rich phases 7
Magnetic scattering of Sr_2CuTeO_6 at various temperatures 8
Integrated inelastic neutron scattering data 9
SPINVERT fits of diffuse magnetic scattering 10
References 11

Crystal structure of $Sr_2CuTe_{0.5}W_{0.5}O_6$ (x = 0.5)

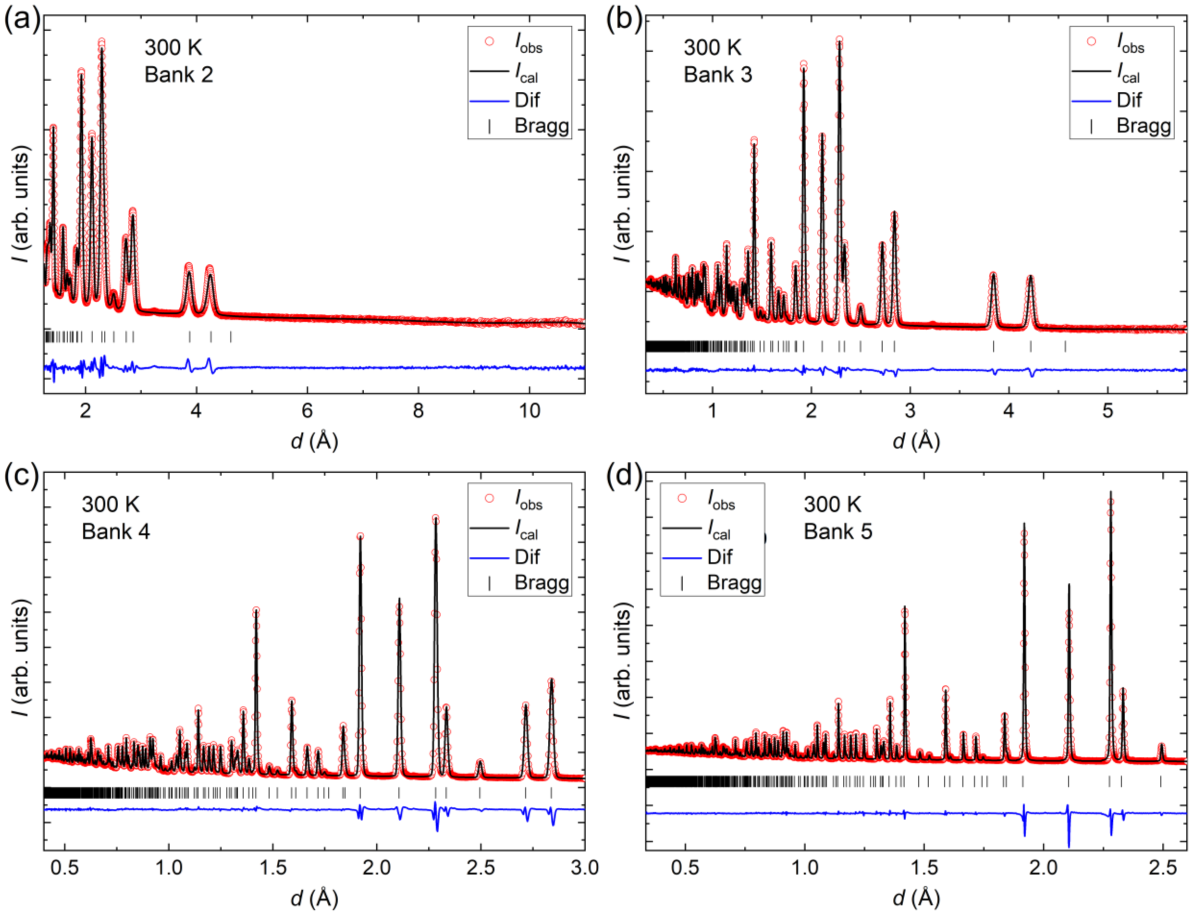

Figure S1. Rietveld refinement of the time-of-flight neutron diffraction data for $Sr_2CuTe_{0.5}W_{0.5}O_6$ (x = 0.5) at 300 K collected on POLARIS. **a** Data for bank 2 at 2ϑ = 25.99° with R_p = 1.55% and R_{wp} = 2.41%. **b** Data for bank 3 at 2ϑ = 52.21° with R_p = 1.26% and R_{wp} = 1.54%. **c** Data for bank 4 at 2ϑ = 92.59° with R_p = 2.23% and R_{wp} = 2.22%. **d** Data for bank 5 at 2ϑ = 146.72° with R_p = 2.19% and R_{wp} = 1.75%.

Table S1. The refined crystal structure of $Sr_2CuTe_{0.5}W_{0.5}O_6$ (x = 0.5) at 300 K based on POLARIS time-of-flight neutron data. Space group $I4/m$ with lattice parameters a = 5.42619(12) Å and c = 8.4239(2) Å. R_p = 2.19% and R_{wp} = 1.75% for the high-resolution bank 5 (2ϑ = 146.72°).

Atom	x	y	z	Occ	U (Å^2)
Sr	0	0.5	0.25	1	$U_{11} = U_{22}$ = 0.0061(2), U_{33} = 0.0077(4)
Cu	0	0	0.5	1	$U_{11} = U_{22}$ = 0.0026(4), U_{33} = 0.0095(6)
Te	0	0	0	0.5	$U_{11} = U_{22}$ = 0.0035(6), U_{33} = 0.0029(8)
W	0	0	0	0.5	$U_{11} = U_{22}$ = 0.0035(6), U_{33} = 0.0029(8)
O1	0.2051(3)	0.2890(2)	0	1	U_{11} = 0.0074(7), U_{22} = 0.0040(7), U_{33} = 0.0133(4), U_{12} = -0.0032(2)
O2	0	0	0.22604(14)	1	$U_{11} = U_{22}$ = 0.0104(3), U_{33} = 0.0046(5)

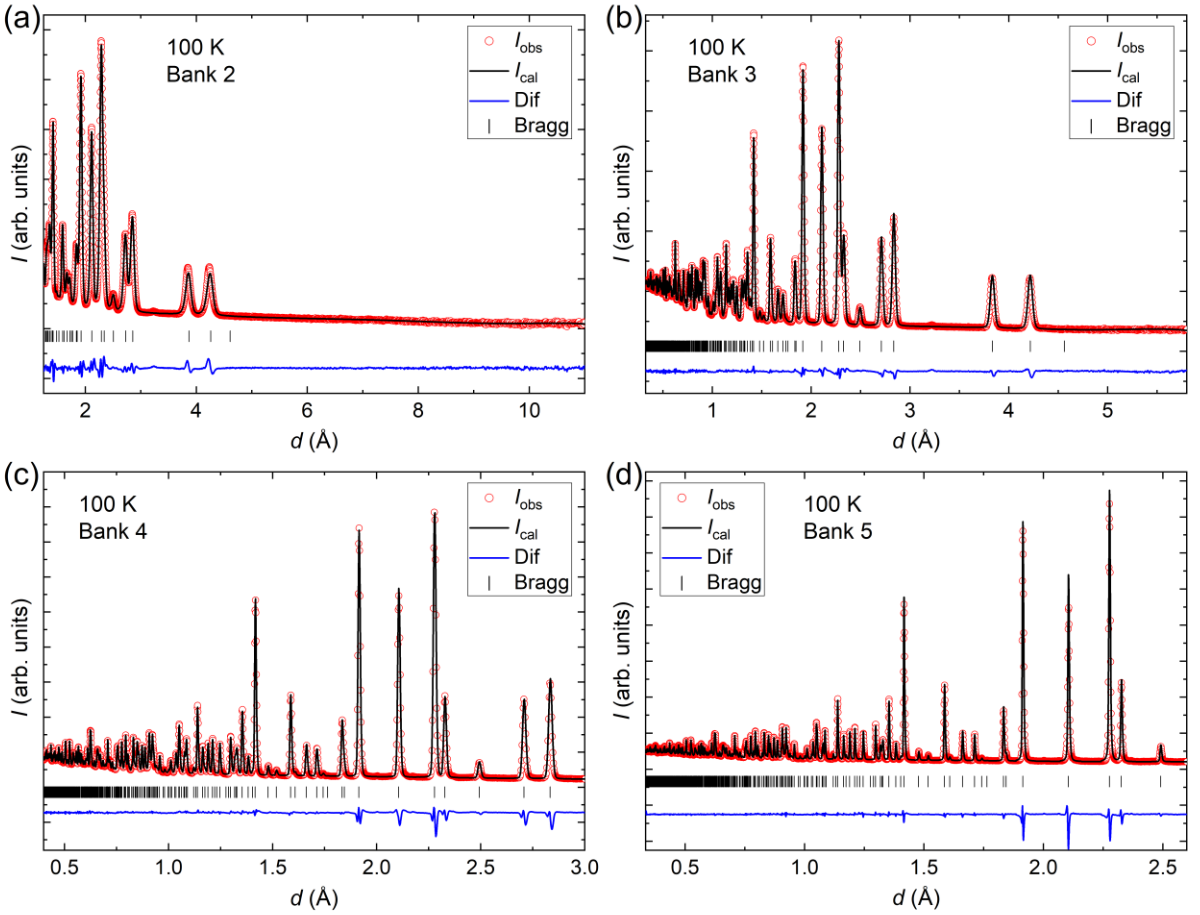

Figure S2. Rietveld refinement of the time-of-flight neutron diffraction data for $Sr_2CuTe_{0.5}W_{0.5}O_6$ (x = 0.5) at 100 K collected on POLARIS. **a** Data for bank 2 at $2\vartheta = 25.99°$ with $R_p = 1.51\%$ and $R_{wp} = 2.33\%$. **b** Data for bank 3 at $2\vartheta = 52.21°$ with $R_p = 1.25\%$ and $R_{wp} = 1.48\%$. **c** Data for bank 4 at $2\vartheta = 92.59°$ with $R_p = 2.36\%$ and $R_{wp} = 2.29\%$. **d** Data for bank 5 at $2\vartheta = 146.72°$ with $R_p = 2.53\%$ and $R_{wp} = 2.01\%$.

Table S2. The refined crystal structure of $Sr_2CuTe_{0.5}W_{0.5}O_6$ ($x = 0.5$) at 100 K based on POLARIS time-of-flight neutron data. Space group *I*4/*m* with lattice parameters $a = 5.41226(10)$ Å and $c = 8.41815(18)$ Å. $R_p = 2.53\%$ and $R_{wp} = 2.01\%$ for the high-resolution bank 5 ($2\vartheta = 146.72°$).

Atom	*x*	*y*	*z*	Occ	*U* (Å^2)
Sr	0	0.5	0.25	1	$U_{11} = U_{22} = 0.0025(2)$, $U_{33} = 0.0029(3)$
Cu	0	0	0.5	1	$U_{11} = U_{22} = 0.0007(3)$, $U_{33} = 0.0051(5)$
Te	0	0	0	0.5	$U_{11} = U_{22} = 0.0018(4)$, $U_{33} = 0.0015(7)$
W	0	0	0	0.5	$U_{11} = U_{22} = 0.0018(4)$, $U_{33} = 0.0015(7)$
O1	0.2020(2)	0.2915(2)	0	1	$U_{11} = 0.0050(5)$, $U_{22} = 0.0021(5)$, $U_{33} = 0.0060(2)$, $U_{12} = -0.0016(2)$
O2	0	0	0.2266(1)	1	$U_{11} = U_{22} = 0.0052(2)$, $U_{33} = 0.0027(4)$

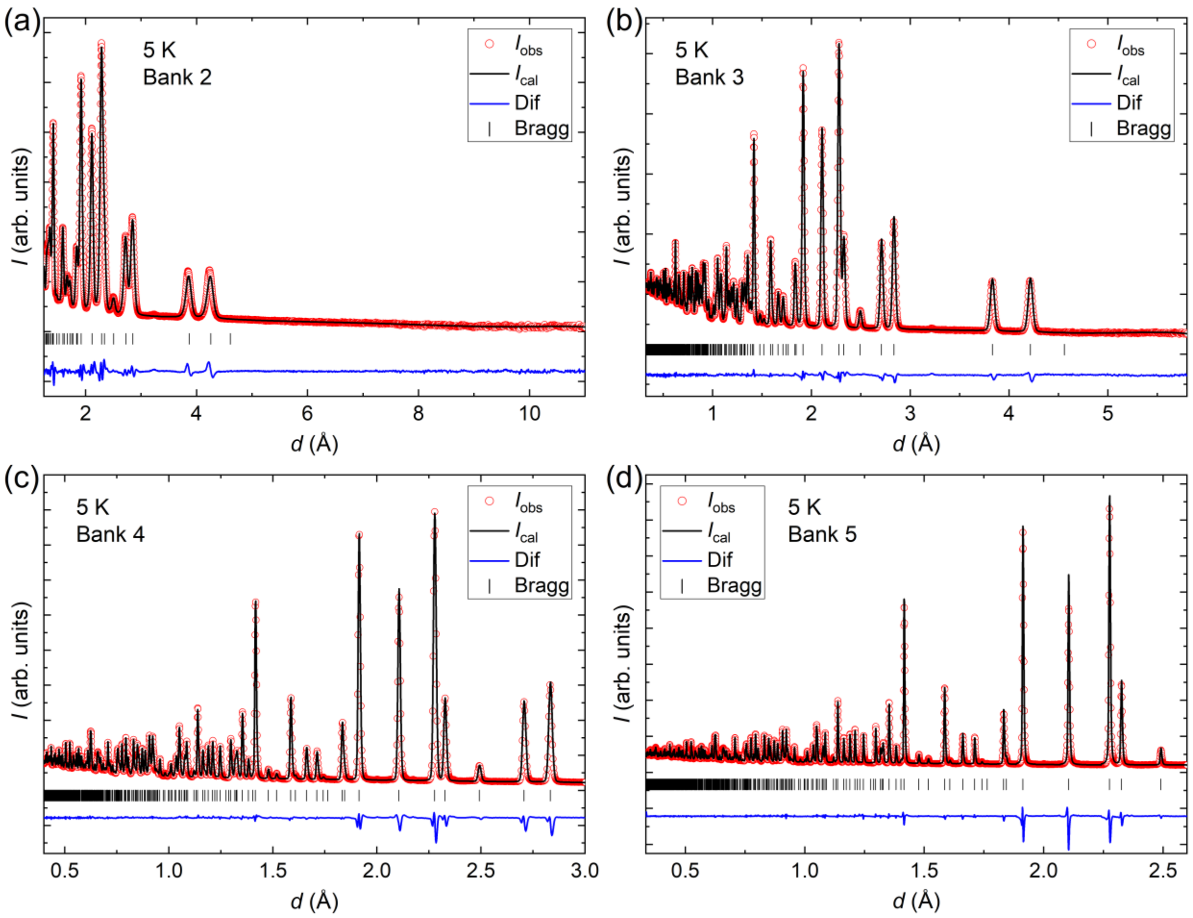

Figure S3. Rietveld refinement of the time-of-flight neutron diffraction data for $Sr_2CuTe_{0.5}W_{0.5}O_6$ (x = 0.5) at 5 K collected on POLARIS. **a** Data for bank 2 at $2\vartheta = 25.99°$ with $R_p = 1.72\%$ and $R_{wp} = 2.39\%$. **b** Data for bank 3 at $2\vartheta = 52.21°$ with $R_p = 1.24\%$ and $R_{wp} = 1.46\%$. **c** Data for bank 4 at $2\vartheta = 92.59°$ with $R_p = 2.38\%$ and $R_{wp} = 2.29\%$. **d** Data for bank 5 at $2\vartheta = 146.72°$ with $R_p = 2.60\%$ and $R_{wp} = 2.05\%$.

Table S3. The refined low-temperature crystal structure of $Sr_2CuTe_{0.5}W_{0.5}O_6$ ($x = 0.5$) at 5 K based on POLARIS time-of-flight neutron data. Space group *I*4/*m* with lattice parameters $a = 5.41025(10)$ Å and $c = 8.41718(18)$ Å. $R_p = 2.60\%$ and $R_{wp} = 2.05\%$ for the high-resolution bank 5 ($2\vartheta = 146.72°$). This table is reproduced here for convenience from Table 1 in the article.

Atom	*x*	*y*	*Z*	Occ	*U* (Å^2)
Sr	0	0.5	0.25	1	$U_{11} = U_{22} = 0.0016(2)$, $U_{33} = 0.0017(3)$
Cu	0	0	0.5	1	$U_{11} = U_{22} = 0.0004(3)$, $U_{33} = 0.0040(5)$
Te	0	0	0	0.5	$U_{11} = U_{22} = 0.0013(4)$, $U_{33} = 0.0012(6)$
W	0	0	0	0.5	$U_{11} = U_{22} = 0.0013(4)$, $U_{33} = 0.0012(6)$
O1	0.2015(2)	0.2917(2)	0	1	$U_{11} = 0.0046(5)$, $U_{22} = 0.0018(5)$, $U_{33} = 0.0047(3)$, $U_{12} = -0.0014(2)$
O2	0	0	0.2267(1)	1	$U_{11} = U_{22} = 0.0043(2)$, $U_{33} = 0.0021(4)$

Refinement of anisotropic *U* parameters for Sr_2CuWO_6

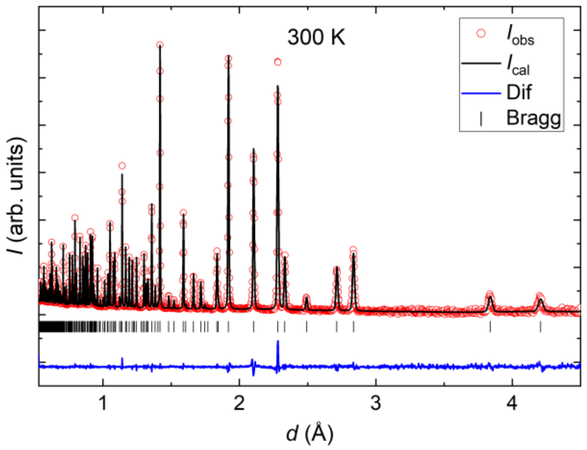

Figure S4. Rietveld refinement of the time-of-flight neutron diffraction data for Sr_2CuWO_6 (x = 1) at 300 K collected on BL-11A POWGEN at Spallation Neutron Source (SNS). This data has been previously published at ref. [1] without refinement of anisotropic *U* parameters. R_p = 4.93% and R_{wp} = 3.08%.

Table S4. The refined crystal structure of Sr_2CuWO_6 (x = 1) at 300 K based on BL-11A POWGEN time-of-flight neutron data. Space group *I*4/*m* with lattice parameters a = 5.42911(4) Å and c = 8.41587(8) Å. R_p = 4.93% and R_{wp} = 3.08%.

Atom	*x*	*y*	*Z*	Occ	*U* (Å^2)
Sr	0	0.5	0.25	1	U_{11} = U_{22} = 0.0075(2), U_{33} = 0.0097(2)
Cu	0	0	0.5	1	U_{11} = U_{22} = 0.0040(3), U_{33} = 0.0093(3)
W	0	0	0	1	U_{11} = U_{22} = 0.0040(3), U_{33} = 0.0039(4)
O1	0.2062(2)	0.2861(2)	0	1	U_{11} = 0.0070(3), U_{22} = 0.0053(3), U_{33} = 0.0158(3), U_{12} = -0.0026(2)
O2	0	0	0.2267(1)	1	U_{11} = U_{22} = 0.0131(2), U_{33} = 0.0037(3)

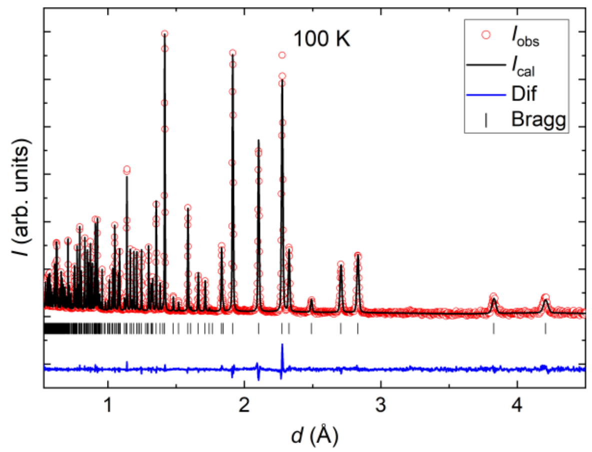

Figure S5. Rietveld refinement of the time-of-flight neutron diffraction data for Sr_2CuWO_6 (x = 1) at 100 K collected on BL-11A POWGEN at Spallation Neutron Source (SNS). This data has been previously published at ref. [1] without refinement of anisotropic U parameters. R_p = 4.93% and R_{wp} = 3.27%.

Table S5. The refined crystal structure of Sr_2CuWO_6 (x = 1) at 100 K based on BL-11A POWGEN time-of-flight neutron data. Space group $I4/m$ with lattice parameters a = 5.41456(4) Å and c = 8.41549(7) Å. R_p = 4.93% and R_{wp} = 3.27%.

Atom	x	y	Z	Occ	U (Å^2)
Sr	0	0.5	0.25	1	U_{11} = U_{22} = 0.0033(2), U_{33} = 0.0038(2)
Cu	0	0	0.5	1	U_{11} = U_{22} = 0.0019(2), U_{33} = 0.0045(3)
W	0	0	0	1	U_{11} = U_{22} = 0.0018(3), U_{33} = 0.0028(4)
O1	0.2029(2)	0.2892(2)	0	1	U_{11} = 0.0043(3), U_{22} = 0.0032(3), U_{33} = 0.0078(2), U_{12} = -0.0014(2)
O2	0	0	0.2273(1)	1	U_{11} = U_{22} = 0.0062(2), U_{33} = 0.0029(2)

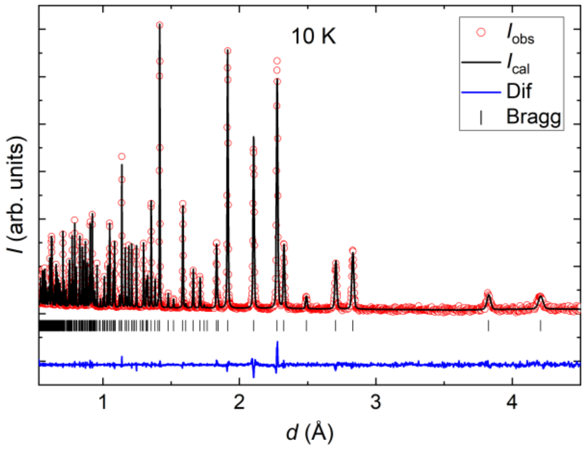

Figure S6. Rietveld refinement of the time-of-flight neutron diffraction data for Sr_2CuWO_6 (x = 1) at 10 K collected on BL-11A POWGEN at Spallation Neutron Source (SNS). This data has been previously published at ref. [1] without refinement of anisotropic U parameters. R_p = 4.98% and R_{wp} = 3.39%.

Table S6. The refined low-temperature crystal structure of Sr_2CuWO_6 (x = 1) at 10 K based on BL-11A POWGEN time-of-flight neutron data. Space group *I*4/*m* with lattice parameters a = 5.41251(4) Å and c = 8.41573(7) Å. R_p = 4.98% and R_{wp} = 3.39%.

Atom	*x*	*Y*	*Z*	Occ	*U* (Å^2)
Sr	0	0.5	0.25	1	$U_{11} = U_{22}$ = 0.0024(2), U_{33} = 0.0028(2)
Cu	0	0	0.5	1	$U_{11} = U_{22}$ = 0.0017(2), U_{33} = 0.0036(3)
W	0	0	0	1	$U_{11} = U_{22}$ = 0.0012(3), U_{33} = 0.0022(4)
O1	0.2024(2)	0.2896(2)	0	1	U_{11} = 0.0039(3), U_{22} = 0.0026(3), U_{33} = 0.0065(2), U_{12} = -0.0012(1)
O2	0	0	0.2274(6)	1	$U_{11} = U_{22}$ = 0.0052(2), U_{33} = 0.0024(3)

Magnetic scattering in the ordered W-rich phases

Table S7. Integrated magnetic Bragg peak areas for the magnetically ordered $Sr_2CuTe_{1-x}W_xO_6$ samples x = 0.9, 0.8 and 0.7. D20 data is shown in main article Figure 5a. The peak area ratio of the (0½0) and (0½½) reflections change with x. This suggests the origin of the additional (½00) reflection is magnetic phase separation with two different propagation vectors **k** = (0, ½, 0) and **k** = (0, ½, ½). If the (0½0) reflection arose as part of a more complicated multi-**k** magnetic structure, the (0½0)/(0½½) ratio would be expected to remain constant.

Sample	(0½0) area	(0½½) area	(0½0)/(0½½)
x = 0.9	5.45	81.56	0.07
x = 0.8	20.33	15.00	1.36
x = 0.7	14.69	12.96	1.13

Magnetic scattering of Sr_2CuTeO_6 at various temperatures

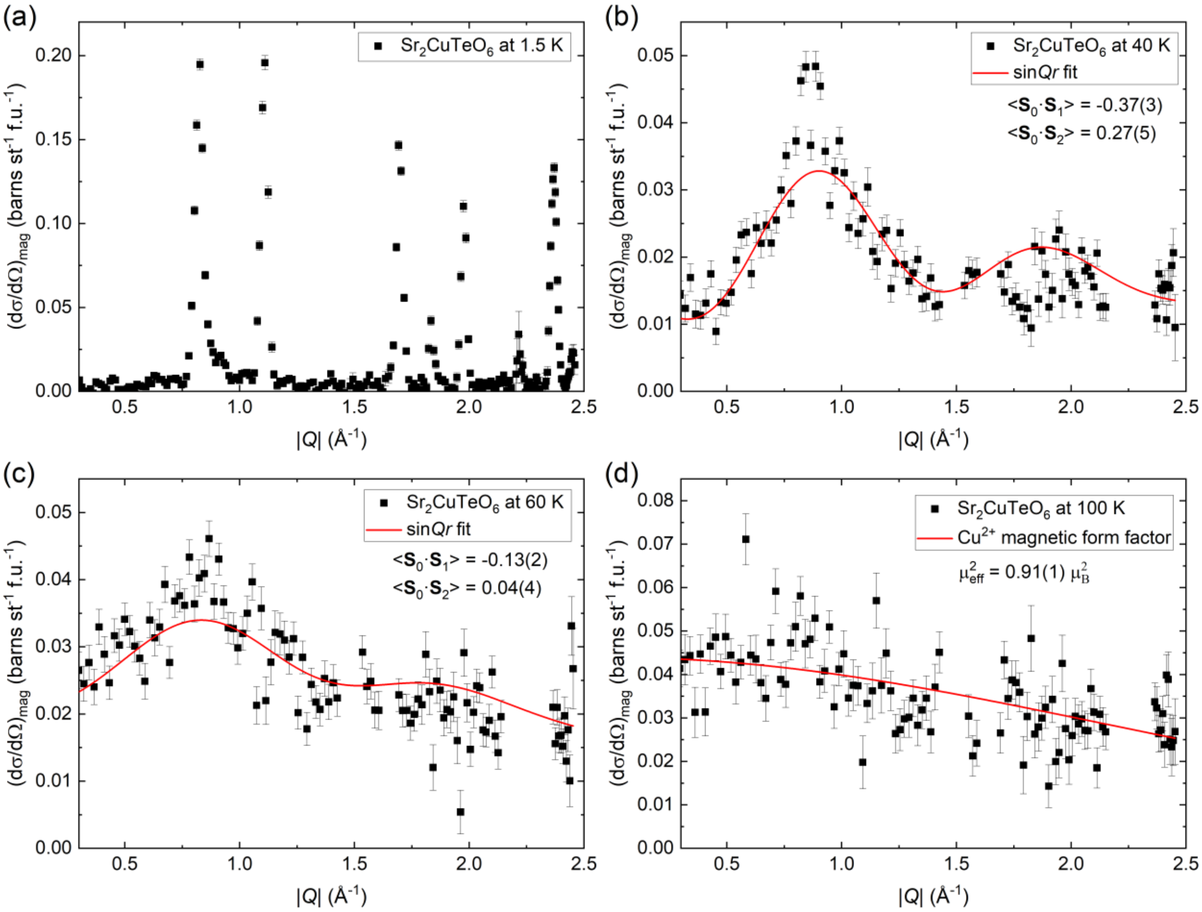

Figure S7. Magnetic scattering of Sr_2CuTeO_6 (x = 0) at **a** 1.5 K, **b** 40 K, **c** 60 K and **d** 100 K. Magnetic Bragg peaks are clearly visible in the 1.5 K data as expected below $T_N \approx 29$ K. Diffuse magnetic scattering indicative of Néel-type correlations is observed at 40 K and 60 K. At 100 K, the scattering is mainly paramagnetic, but minor correlations appear to be still present around $|Q|$ = 0.85 Å^{-1}. A fit to the Cu^{2+} magnetic form factor yields μ_{eff}^2 = 0.91(1) μ_{B}^2, which is a third of the expected value. Therefore, our experiment captures only the lowest 1/3rd energy excitations in these compounds.

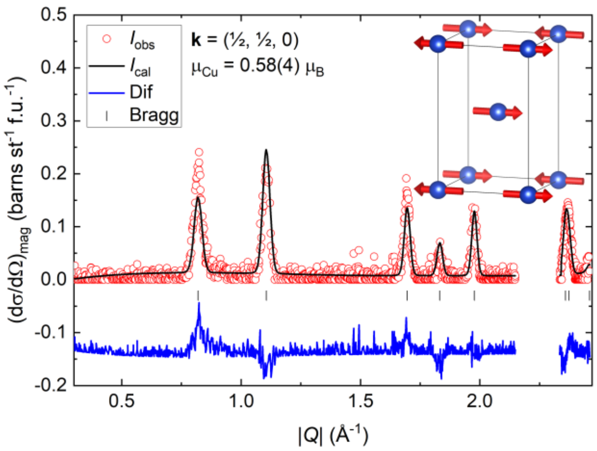

Figure S8. Refined magnetic structure of Sr_2CuTeO_6 at 1.5 K. Propagation vector **k** = (½, ½, 0), a moment of 0.58(4) μ_B per copper and R_{mag} = 23.5 %. The refined moment is slightly smaller than the 0.69(6) μ_B previously reported in ref. [2], but consistent with the 0.57(1) μ_B reported for Sr_2CuWO_6.[3] Inset: The Néel antiferromagnetic structure of Sr_2CuTeO_6. The nearest-neighbor spins order antiferromagnetically along the side of the square, while the in-plane next-nearest-neighbor (square diagonal) and interplane ordering along *c* are ferromagnetic.

Integrated inelastic neutron scattering data

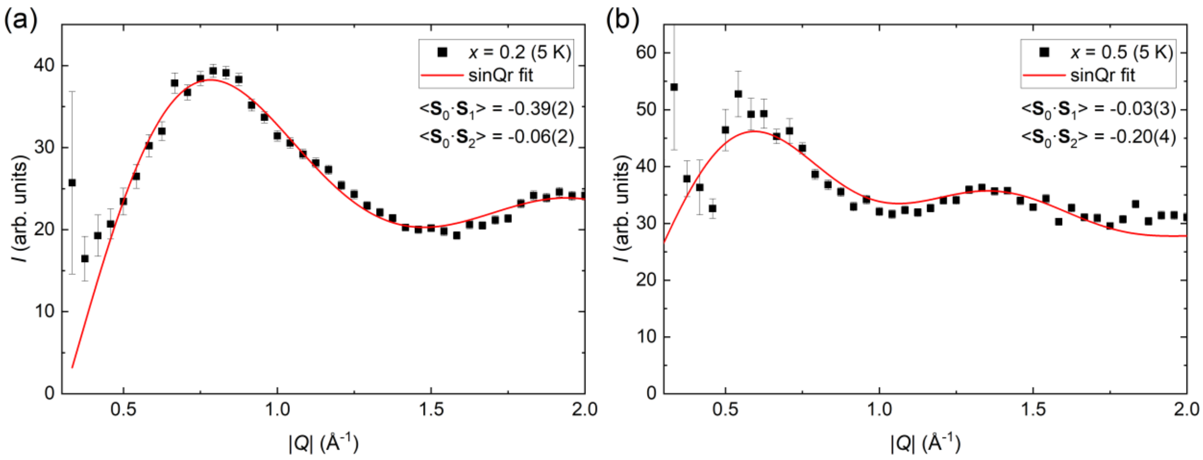

Figure S9. Integrated inelastic neutron scattering between 2.5 meV and 7.5 meV energy transfer for $Sr_2CuTe_{1-x}W_xO_6$ samples **a** x = 0.2 and **b** x = 0.5. Data from refs. [4,5] collected on MERLIN at T = 5 K with E_i = 18 meV. The positions of the maximums and the overall shapes of the curves are very similar to the D7 measurements with E_i = 3.55 meV. This shows that despite not capturing the full spectral weight, the D7 data is representative of the overall scattering at least up to 7.5 meV. The limited low-|Q| coverage of the MERLIN data at higher energies prohibits comparisons up to 15-20 meV, where features are still observed in the spectra.

SPINVERT fits of diffuse magnetic scattering

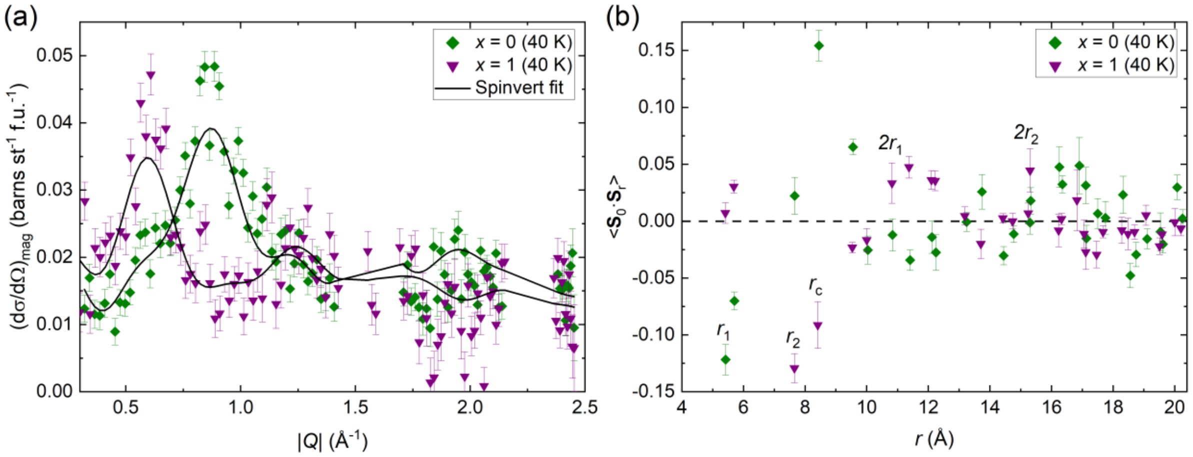

Figure S10. **a** SPINVERT fits of the diffuse magnetic scattering of Sr_2CuTeO_6 (x = 0) and Sr_2CuWO_6 (x = 1) at 40 K in the short-range correlated state above T_N. The quality of the fits is limited by the very weak magnetic scattering and limited energy coverage. The magnetic scattering of x = 0 has a peak at $|Q| \approx 0.85$ Å^{-1}, which is related to the (½½0) reflection of the Néel magnetic structure below T_N. For x = 1, a peak is observed at $|Q| \approx 0.6$ Å^{-1}, which is related to the main (0½½) magnetic Bragg peak of the columnar structure and inelastic scattering from the forbidden (0½0) reflection due to the weak interlayer coupling. **b** Spin correlation functions of Sr_2CuTeO_6 and Sr_2CuWO_6 obtained from SPINVERT fits. The spin correlations for x = 0 are Néel-type with antiferromagnetic $\langle \boldsymbol{S}_0 \cdot \boldsymbol{S}_1 \rangle$ correlations and ferromagnetic $\langle \boldsymbol{S}_0 \cdot \boldsymbol{S}_2 \rangle$ correlations. The correlations in x = 1 are columnar-type with very weak $\langle \boldsymbol{S}_0 \cdot \boldsymbol{S}_1 \rangle$ and antiferromagnetic $\langle \boldsymbol{S}_0 \cdot \boldsymbol{S}_2 \rangle$ correlations.

Table S8. In-plane spin correlations obtained from the SPINVERT fits of $Sr_2CuTe_{1-x}W_xO_6$ diffuse magnetic scattering and the expected spin correlations for complete Néel or columnar magnetic order. r_1 corresponds to the side of the square, r_2 to the diagonal, r_3 to a Chess Knight move (two lengths along one side and one along a perpendicular side) and r_c to the interlayer distance.

	r_i (Å)	Z_i	Néel	Columnar	x = 0	x = 0.2	x = 0.5	x = 1
r_1	5.4	4	-1	0	-0.12(1)	-0.12(1)	-0.01(2)	0.01(1)
r_2	7.6	4	1	-1	0.02(1)	0.07(1)	-0.12(1)	-0.13(1)
r_c	8.4	2	1	-1	0.15(2)	0.05(2)	-0.04(2)	-0.09(2)
$2r_1$	10.8	4	1	1	-0.01(1)	0.02(1)	0.08(2)	0.03(2)
r_3	12.1	8	-1	0	-0.01(1)	-0.01(1)	0.02(1)	0.04(1)
$2r_2$	15.3	4	1	1	0.0(1)	-0.01(1)	0.05(2)	0.04(2)

References

(1) Vasala, S.; Saadaoui, H.; Morenzoni, E.; Chmaissem, O.; Chan, T.; Chen, J.; Hsu, Y.; Yamauchi, H.; Karppinen, M. Characterization of Magnetic Properties of Sr_2CuWO_6 and Sr_2CuMoO_6. *Phys. Rev. B* **2014**, *89*, 134419.

(2) Koga, T.; Kurita, N.; Avdeev, M.; Danilkin, S.; Sato, T. J.; Tanaka, H. Magnetic Structure of the S = 1/2 Quasi-Two-Dimensional Square-Lattice Heisenberg Antiferromagnet Sr_2CuTeO_6. *Phys. Rev. B* **2016**, *93*, 054426.

(3) Vasala, S.; Avdeev, M.; Danilkin, S.; Chmaissem, O.; Karppinen, M. Magnetic Structure of Sr_2CuWO_6. *J. Phys. Condens. Matter* **2014**, *26*, 496001.

(4) Katukuri, V. M.; Babkevich, P.; Mustonen, O.; Walker, H. C.; Fåk, B.; Vasala, S.; Karppinen, M.; Rønnow, H. M.; Yazyev, O. V. Exchange Interactions Mediated by Non-Magnetic Cations in Double Perovskites. *Phys. Rev. Lett.* **2020**, *124*, 077202.

(5) Fogh, E.; Mustonen, O.; Babkevich, P.; Katukuri, V. M.; Walker, H. C.; Mangin-Thro, L.; Karppinen, M.; Ward, S.; Normand, B.; Rønnow, H. M. Randomness and Frustration in a S = 1/2 Square-Lattice Heisenberg Antiferromagnet. *Phys. Rev. B* **2022**, *105*, 184410.
